# Phytochemical Fingerprinting of *Sanchezia nobilis* Hook. F. Leaf Extract and Evaluation of Anxiolytic, Cytotoxic, and Antioxidant Activities In Vivo, In Vitro, and In Silico

**DOI:** 10.1002/cbdv.71776

**Published:** 2026-10-09

**Authors:** Progga Paramita Paul, Musfiqur Rahman Sakib, Pritam Kundu, Dabashis Chakraborty, Tasnim Fatema, Shahriar Hossen, Ahsan Abid, Trina Mitra, Manik Chandra Shill, Utpal Kumar Karmakar

**Affiliations:** ^1^ Pharmacy Discipline Life Sciences School Khulna University Khulna Bangladesh; ^2^ Department of Pharmaceutical Sciences North South University Dhaka Bangladesh; ^3^ Department of Pharmacy Jashore University of Science and Technology Jashore Bangladesh

**Keywords:** anxiolytic activity, drug discovery, GABAA receptor, GC‐MS fingerprint, phytochemicals, *Sanchezia nobilis*

## Abstract

Present studies emphasize the pharmacological, toxicological, and molecular simulation studies of the potent phytoconstituents in the *Sanchezia nobilis* leaf extract (SNE) and its nonpolar and polar fractions. We identified 27 phytoconstituents in SNE using Gas Chromatography Mass Spectrometry (GC‐MS), where aniline, dibutyl phthalate, and isoquinoline are noteworthy. Anxiolytic assessments by hole board test (HBT) and elevated plus maze test (EPMT) demonstrated dose‐dependent effects of nonpolar and polar fractions of SNE. Both fractions also exhibited minimal cytotoxicity in brine shrimp lethality test. SNE crude extract demonstrated good antioxidant properties. In silico analysis revealed promising binding affinities of aniline and isoquinoline with target protein molecules responsible for anxiolytic and antidepressive effects. Molecular dynamics (MD) modelling and quantitative chemical‐based DFT approaches confirmed satisfactory pharmacokinetics (PK) and toxicity profiles for the selected medications. HOMO‐LUMO analysis highlighted the remarkable features of the chosen components. MD studies also supported aniline and isoquinoline as potential candidates requiring further pharmacological validation due to their interactions with chimeric alpha1GABA_A_ receptor proteins. Our data provided promising drug‐like phytoconstituents in the *S. nobilis* leaf offering future drug discovery in mental health treatment.

## Graphical abstract

GC‐MS fingerprinting demonstrated that ethanolic leaf extract of *Sanchezia nobilis* (SNE) many phytochemicals out of which aniline and isoquinoline showed potential binding affinity to alpha1GABA_A_ receptor responsible for anxiolytic effects. In vitro studies confirmed antioxidant effects of SNE. In vivo studies showed significant anxiolytic effects of the polar and nonpolar fractions of the extract.

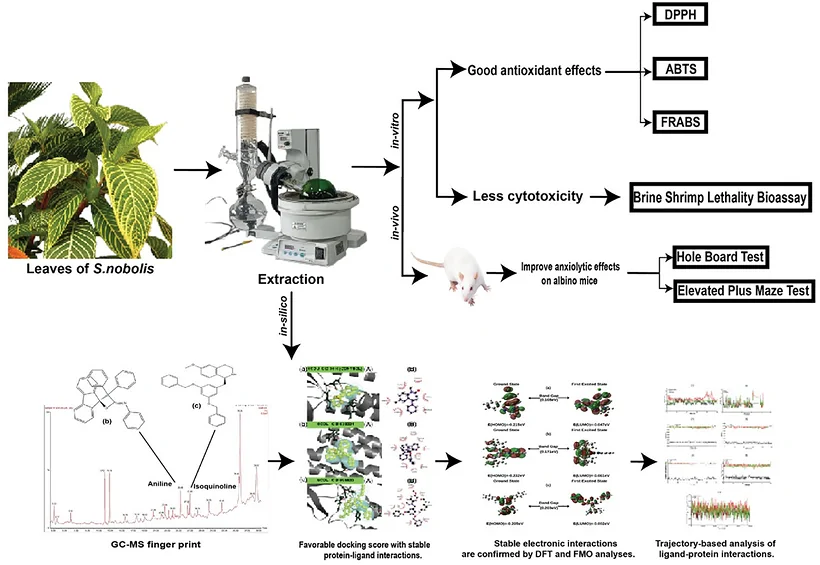

## 1 Introduction

Naturehas served as a fundamental source of medicine from the inception of human civilization. Natural compounds having antioxidant properties have become a prominent focus of health research due to their potentials to prevent or delay oxidative damage linked to a number of chronic diseases. Damage from oxidative stress is a prevalent sign of neurodegenerative diseases. The study of organic antioxidants is one of the primary challenges to developing new therapies from natural resources. The ability to scavenge reactive oxygen species (ROS), makes natural antioxidants a promising therapeutic strategy for neurological illnesses such as amyotrophic lateral sclerosis (ALS), Parkinson's disease, Alzheimer's disease, Huntington's disease, as well as dementia. Drug discovery using plant resources is still a significant source of novel lead molecules, but there are many obstacles to overcome [1].

Plants are the prime source of bioactive compounds for medical care for a great proportion of the population of the developing world. Plants generally produce secondary metabolites in response to environmental stress. The treatment of human diseases with herbal products has several advantages due to availability, affordability, low level of side effects and so forth. Furthermore, natural products have enormous untapped potential for finding novel bioactive compounds for medical applications [2]. The perennial evergreen shrub *Sanchezia nobilis* (Acanthaceae) is indigenous to central and southern America's tropical rainforests. *Sanchezia* species have been employed traditionally for a multitude of therapeutic purposes, comprising linctus, coughing sedative, antituberculosis, anticancer, and anticonvulsant [3]. This plant possesses benzyl alcohol, cinnamyl alcohol, flavonoid glycosides, matsutake alcohol glycosides, daucosterol, stigmasterol, '5,7‐trihydroxy‐3',5'‐ dimethoxyflavone, kaempferol‐3‐O‐α‐L‐arabinofuranoside, and kaempferol‐3‐O‐β‐D‐glucopyranoside [4]. It has been reported that, *S. nobilis* leaf extract has analgesic, anthelmintic, antidiarrheal, and central nervous system (CNS) depressive properties [5]. Recent research points to a strong connection between oxidative stress and anxiety disorders [6]. Oxidative stress disrupts normal functions of the brain contributing to symptoms like anxiety [7]. Compounds with antioxidant effects may help protect brain cells from this oxidative damage and reduce anxiety. *S. nobilis*, a plant known to have antioxidant content, could therefore, be a useful natural option for managing anxiety [8]. Active components of the plant may reduce oxidative harm, which in turn modulate GABA neurotransmitter system to provide neurological effect [9]. *S. nobilis* is a rare plant in Bangladesh, and so far, few research has been conducted in Bangladesh. As a part of ongoing exploration of Bangladeshi plants with medicinal values, our primary focus was to identify chemical constituents present in the leaf of *S. nobilis* using GC‐MS, and examine the antioxidant and cytotoxic activity. Further studies emphasized on the anxiolytic effects of the nonpolar and polar fraction of SNE in light of the previous research. The study also aimed to find out the optimal ligands as promising pharmacological candidates through in silico research.

Molecular docking and simulation studies are of great interest that not only support biological data but also play significant role in reducing cost and time of drug discovery [10, 11]. All the computer‐based methods like molecular docking and dynamics simulations are now widely used to anticipate drug like properties of natural compounds [12]. These tools can help researchers in screening plant chemicals to bind with important proteins, which give clues about their targeted health benefits. A combined wet lab experiments and simulations provide a wider mapping of a compound in treating diseases [13, 14]. So, aim of the study is to identify leads responsible for the biological activity supported by computational approaches.

## 2 Results and Discussions

### 2.1 Evaluation of Anxiolytic Activity

In the Hole Board Test (HBT) test, both polar and nonpolar fractions of SNE showed substantial anxiolytic activity denoted by reducing the no. of head dipping over the period of time. At higher dose (500 mg/kg) both fractions exhibited more than 60% activity which is close to the standard drug. We also found significant anxiolytic activity of both the polar and nonpolar fractions of SNE in elevated plus maze test (EPMT) test. At higher dose (500 mg/kg) both fractions showed better anxiolytic effect which is comparable to the standard drug diazepam (open and close arms test). The results of HBT and EPMT are presented in Figure 1.

**FIGURE 1:**
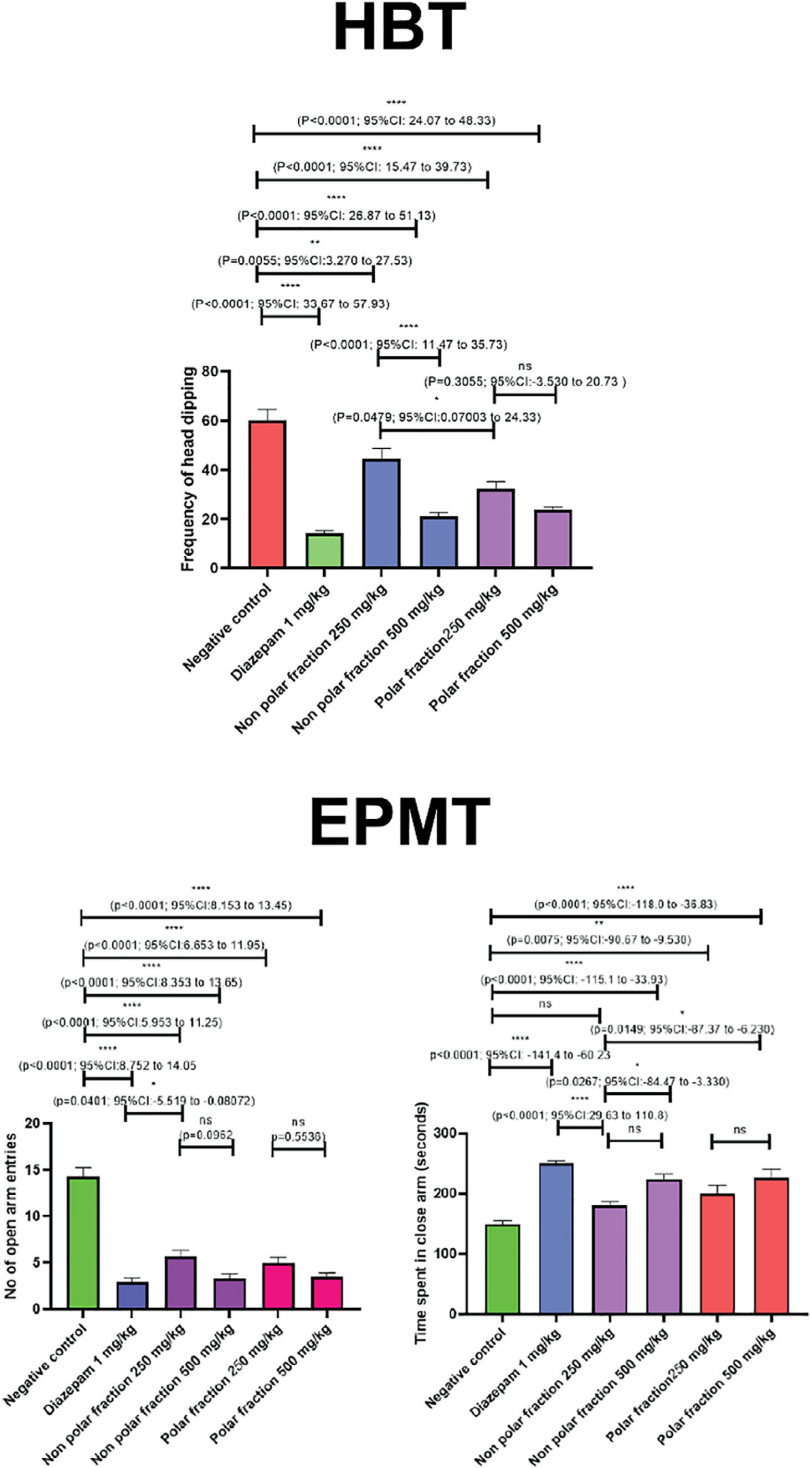
Effects of the polar and nonpolar fractions of SNE on exploratory behavior (hole‐board test) and anxiolytic activity (elevated plus‐maze test) in Swiss albino mice. Data is represented as mean ± SEM. Adjusted p value and 95%CI are provided to clarify the data significance. *p < 0.05; **p < 0.01; ***p < 0.001; ****p < 0.0001 (*n* = 10). Nonpolar fraction: Hexane fraction; polar fraction: water fraction.

### 2.2 Brine Shrimp Lethality Bioassay

Both nonpolar and polar fractions of SNE showed very low cytotoxic activity LC_50_ 112.136 and 100.30 µg/mL respectively compared to the standard (vincristine sulfate) LC_50_ = 0.282 µg/mL Figure 2, explicit the mortality of brine shrimp for both fractions of SNE and vincristine sulphate.

**FIGURE 2:**
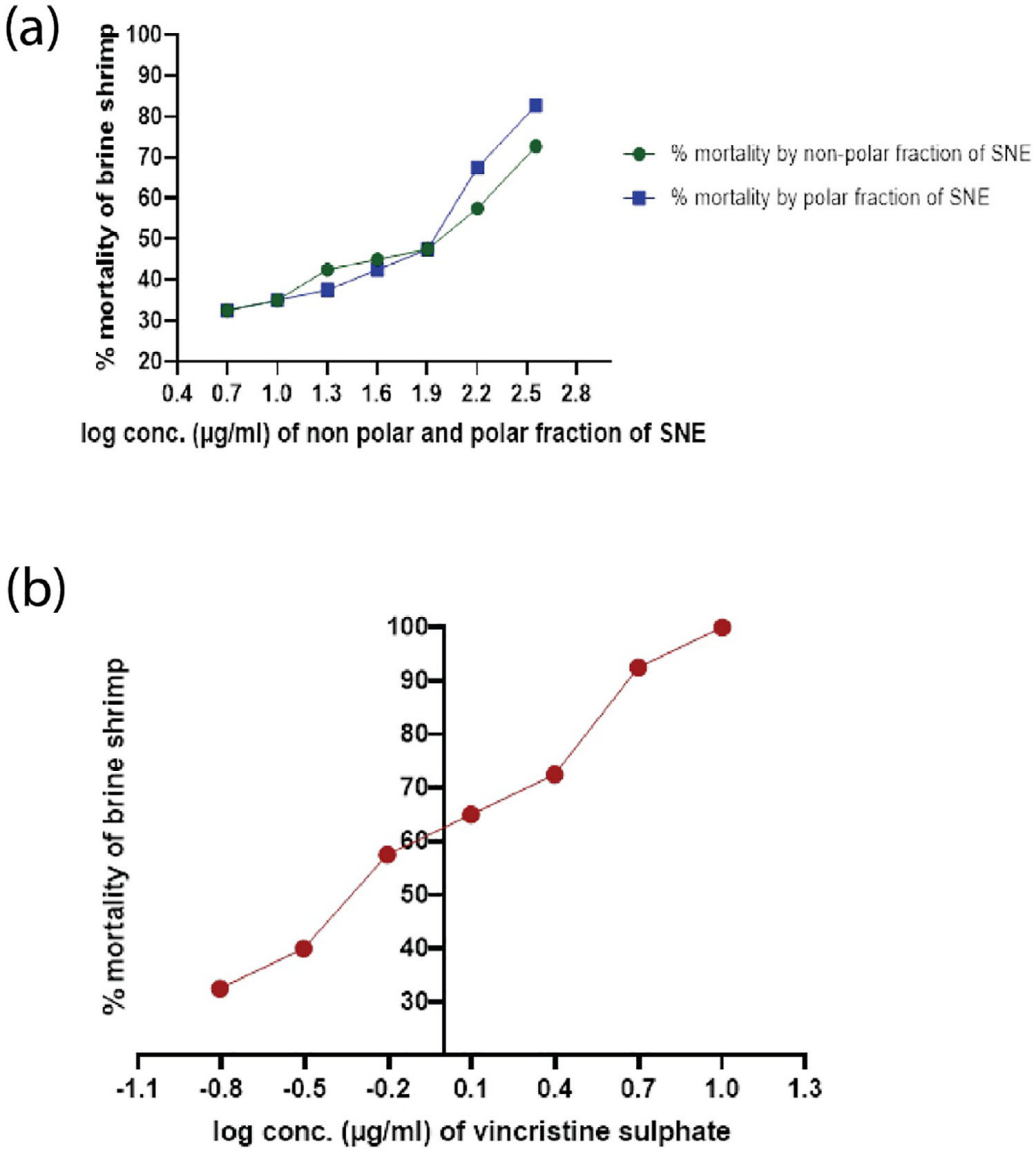
% mortality of brine shrimp for (a) both polar and nonpolar fractions of SNE and (b) vincristine sulphate. Nonpolar fraction: Hexane fraction; polar fraction: water fraction.

### 2.3 Evaluation of Antioxidant Activity

#### 2.3.1 DPPH Assay

The presence of unpaired electron in the DPPH radical showed characteristic absorption when radical accepts electron indicating radical scavenging efficacy. Because of the antioxidant property of the extract, it donates a free electron into the solution, and bind with DPPH resulting in loss of color. From Table 1, we interpret that the inhibition percentage is increasing with the increasing concentration. Crude SNE showed 81.13% scavenging effects at the 1 mg/mL concentration which is comparable with ascorbic acid (92.92%) at the same concentration.

**TABLE 1:** Antioxidant capacity (DPPH assay) of SNE and the standard. Data were taken at different concentrations three times and was presented as Mean ± SEM. We repeat the experiment three times.

Concentration (mg/mL)	% inhibition by the extract (Mean ± SEM)	% inhibition by the standard (Mean ± SEM)
0.015	63.090 ± 0.409	64.38 ± 0.355
0.032	64.42 ± 0.373	66.82 ± 0.885
0.062	72.09 ± 0.549	86.47 ± 0.885
0.125	73.66 ± 0.348	88.4 ± 0.531
0.25	74.8 ± 0.342	91.82 ± 0.594
0.5	76.61 ± 0.306	92.25 ± 0.765
1	80.84 ± 0.271	92.96 ± 0.765

#### 2.3.2 ABTS Assay

After potassium persulfate is added to the ABTS solution, bluish green color was observed. This color change occurs because ABTS loses an electron from its nitrogen atom. In the presence of antioxidant, the nitrogen atom binds with hydrogen atom resulting in decolorization of the solution. From Table 2, it is observable that at highest concentration 6 mg/mL, the inhibition percentage is 80.66% which is close to standard drug (94.78%), means at higher concentration the extract shows greater electron scavenging effect.

**TABLE 2:** Antioxidant capacity (ABTS assay) of SNE and the standard. Data were taken at different concentrations three times and was presented as Mean ± SEM. We repeat the experiment three times.

Concentration (mg/mL)	% inhibition by the extract (Mean ± SEM)	% inhibition by the standard (Mean ± SEM)
1	53.73 ± 0.231	68.43 ± 0.191
2	60.16 ± 0.317	76.59 ± 0.265
3	63.27 ± 0.231	86.88 ± 0.162
4	72.13 ± 0.178	90.38 ± 0.124
5	77.72 ± 0.248	92.22 ± 0.159
6	80.64 ± 0.265	94.78 ± 0.213

#### 2.3.3 FRAP Assay

In FRAP assay, oxidant Fe^3+^ binds with the antioxidant and produce blue colored Fe^2+^. The purpose of FRAP test is to measure the extent of the antioxidant's ferric ion reducing ability. If the absorbance is higher, the antioxidant has more reducing power. From Table 3, we can see that the absorbance value is proportional to concentration which means the extract has antioxidant properties.

**TABLE 3:** Antioxidant capacity (FRAP assay) of SNE and the standard. Data were taken at different concentrations three times and was presented as Mean ± SEM. We repeat the experiment three times.

Concentration (mg/mL)	Absorbance (extract)	Absorbance (standard)
0.1	0.126 ± 0.0028	0.457 ± 0.0026
0.2	0.185 ± 0.0034	0.481 ± 0.0043
0.3	0.222 ± 0.0043	0.578 ± 0.002
0.4	0.287 ± 0.0032	0.663 ± 0.004
0.5	0.36 ± 0.0037	0.7 ± 0.0034
0.6	0.421 ± 0.0023	0.748 ± 0.0063
0.7	0.448 ± 0.0066	0.796 ± 0.0027
0.8	0.598 ± 0.001	0.849 ± 0.0035
0.9	0.668 ± 0.0052	0.98 ± 0.0115
1	0.738 ± 0.0037	1.105 ± 0.0071

### 2.4 GC‐MS Analysis

GC‐MS analysis identified 27 compounds in the SNE crude extract. The GC‐MS spectrum of SNE is plotted in Figure 3. Table 4, displays the retention times, compounds' names, molecular formula, chemical classes, molecular weight, and the compounds' percentage peak areas.

**FIGURE 3:**
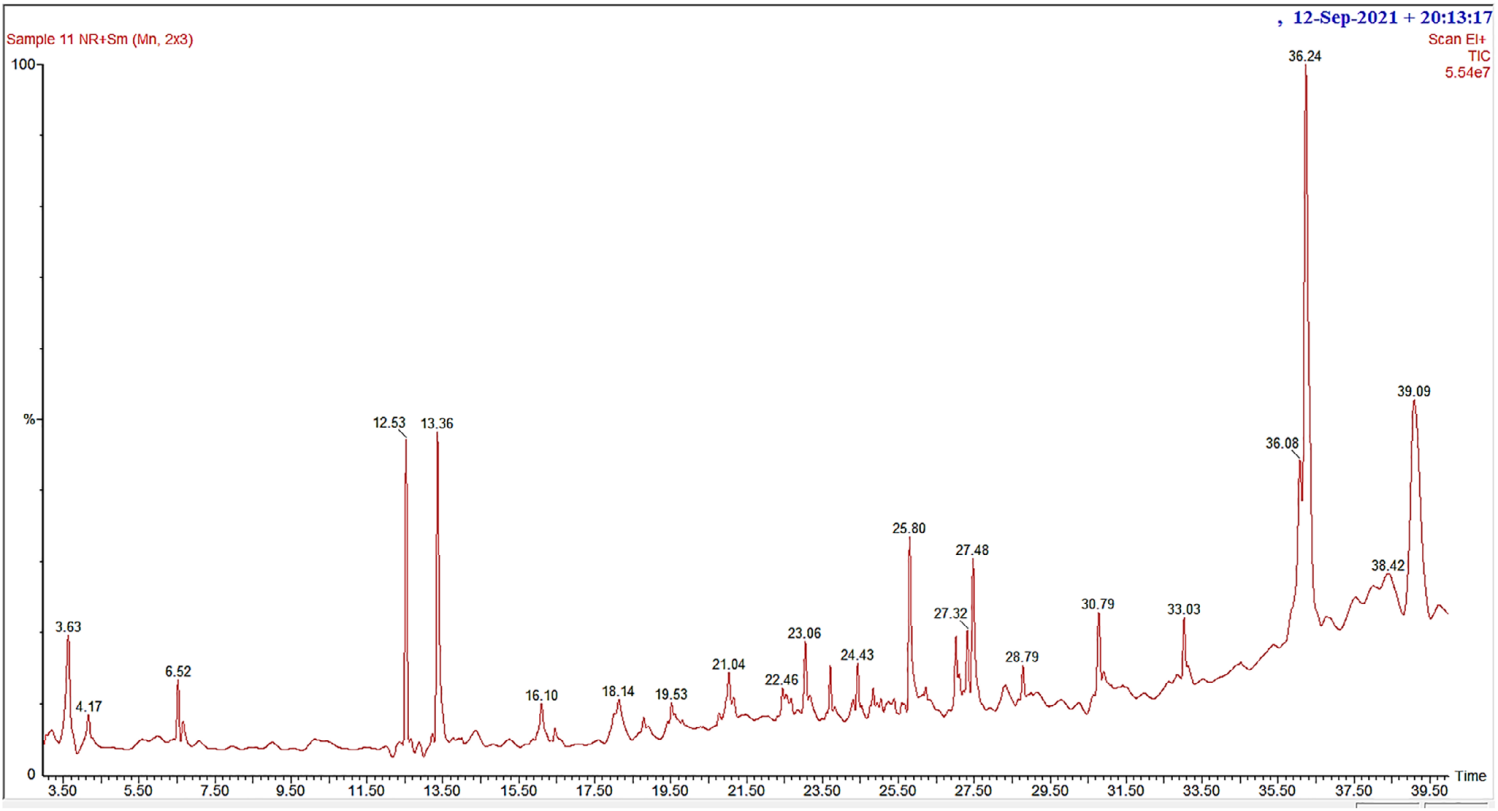
Gas chromatography mass‐spectroscopic (GC‐MS)‐base chromatogram of SNE.

**TABLE 4:** Compounds found in SNE using GC‐MS study. We have identified the compounds by comparing the retention time and molecular weight with the corresponding NIST library data.

Serial No.	Retention time	Name of the compounds	Molecular formula	Chemical class	Molecular weight (gm/mol)	% Peak area
**1**	**3.630**	**Benzene, 1,3‐dimethyl**	**C_8_H_10_**	**Benzenoid** (NP)	**106**	**2.404**
2	4.173	4,6‐octadiyn‐3‐one, 2‐methyl	C_9_H_10_O	Alkyne	134	1.329
3	6.525	Dodecane, 1‐fluoro	C_12_H_25_F	Alkane (NP)	188	1.141
4	6.666	O‐cymene	C_10_H_14_	monoterpenoid	134	0.565
**5**	**12.537**	**Naphthalene**	**C_10_H_8_**	**Bicyclic benzenoid**	**128**	**3.811**
**6**	**13.361**	**Benzaldehyde, 4‐methyl**	**C_8_H_8_O**	**Aldehyde**	**120**	**5.168**
7	16.102	Benzenesulfonohydrazide, n2‐(2‐ethoxybenzylideno)‐4‐methyl	C_16_H_18_O_3_N_2_S	sulfonohydrazides	318	1.153
8	16.458	Hentriacontane	C_31_H_64_	Alkane	436	0.392
9	18.147	Propanoic acid, 3‐chloro‐, 4‐formylphenyl ester	C_10_H_9_ClO_3_	Carboxylic acid (NP)	212	1.774
10	19.527	Vanillin	C_8_H_8_O_3_	Aldehyde	152	1.814
**11**	**21.042**	**Pentyl tetratriacontyl ether**	**C_39_H_80_O**	**Ether**	**564**	**3.555**
12	22.463	1,4‐methanobenzocyclodecene, 1,2,3,4,4a,5,8,9,12,12a‐decahydro	C_15_H_22_	Alkane	202	1.886
**13**	**23.059**	**Bicyclo [3.1.0] hexan‐2‐one, 1,5‐bis(1,1‐dimethylethyl)‐3,3‐dimethyl**	**C_16_H_28_O**	**Alkane**	**236**	**2.411**
14	23.716	Diethyl phthalate	C_12_H_14_O_4_	Carboxylic acid	222	1.383
15	24.314	6‐bromohexanoic acid, 3‐methylbutyl ester	C_11_H_21_O_2_Br	Ester	264	1.412
16	24.428	Neophytadiene	C_20_H_38_	Diterpene	278	1.412
17	24.837	Benzophenone	C_13_H_10_O	Ketone	182	1.120
**18**	**25.811**	**Aniline, n‐(3',3'‐diphenylspiro[fluorene‐9,2'‐oxetan]‐4'‐ylidene)**	**C_33_H_23_NO**	**Amine**	**449**	**3.115**
19	26.228	Benzaldehyde, 4‐hydroxy‐3,5‐dimethoxy	C_9_H_10_O	Aldehyde	182	1.353
20	27.020	Phosphine oxide, bis(pentamethylphenyl)	C_22_H_30_OP^+^	Phosphine	342	1.490
21	27.322	Methyl 11‐methyl‐dodecanoate	C_14_H_28_O_2_	Ester	228	1.074
**22**	**27.483**	**Isoquinoline, 1‐[(3,5‐dibenzyloxy) benzyl]‐1,2,3,4‐tetrahydro‐6‐methoxy**	**C_31_H_31_O_3_N**	**Alkaloid**	**465**	**2.284**
23	28.329	Benzoic acid, 4‐hydroxy‐3,5‐dimethoxy‐, hydrazide	C_9_H_12_N_2_O	Carboxylic acid	212	1.076
24	28.793	1,2‐benzenedicarboxylic acid, bis(2‐methylpropyl) ester	C_16_H_22_O	Carboxylic acid	278	0.895
25	30.787	Dibutyl phthalate	C_16_H_22_O_4_	Benzoic acid ester	278	4.087
26	33.037	2‐propenal, 3‐(3,4‐dimethoxyphenyl)	C_11_H_12_O	Aldehyde	192	1.262
**27**	**36.241**	**1,4‐benzenedicarboxylic acid, bis(2‐ethylhexyl) ester**	**C_24_H_38_O_4_**	**Carboxylic aid**	**390**	**18.755**

### 2.5 In Silico Analysis

#### 2.5.1 Pharmacokinetics Analysis

To assess the pharmacokinetic attributes of the reference drug and principal phytoconstituents identified in the extract, diazepam, [n‐(3',3'‐diphenylspiro[fluorene‐9,2'‐oxetan]‐4'‐ylidene)] and 1‐[(3,5‐dibenzyloxy) benzyl]‐1,2,3,4‐tetrahydro‐6‐methoxy) isoquinoline are selected for analysis. The molecular masses of the selected compounds are 284.74, 449.54, and 465.58 g/mol respectively. Among the compounds, aniline exhibited highest number of Lipinski rule‐of‐five violations (two). Aniline also demonstrated bioavailability score 0.55 and no AMES mutagenicity demonstrating its satisfactory safety profile. Comparative analysis provided least topographically polarized contact diameter of the major phytoconstituents compared to reference drug suggesting better membrane permeability of the leads. None of the studied compounds demonstrated hepatotoxicity. The maximum tolerated doses (log mg/kg/day) of the compounds in human have been found in between 0.066 and 0.446, while the lethal dose (LD_50_) was found to be approximately 3.00 which indicate its adequate therapeutic margin. The octanol/water partitioning coefficient (Log P) for the investigated leads ranged from 2.97 to 6.75 (log mL/min/kg) demonstrating medium to high lipophilicity and better membrane diffusion. Whereas the experimental total clearance (TC) values were ranges from 0.294 to 0.996 (log mL/min/kg) indicating moderate rate of elimination. Table 5, explicitly shows rotatable bonds, hydrogen bond donors, and hydrogen bond acceptors, along with additional factors.

**TABLE 5:** Pharmacokinetics analysis of the compounds diazepam, aniline, and isoquinoline.

Parameters	Diazepam (Control)	Aniline	Isoquinoline
**Mwe (g/mol)** ^a^	284.74	449.54	465.58
**HBA** ^b^	2	2	4
**HBD** ^c^	0	0	1
**Num. rot** ^d^	1	3	9
**BBB** ^e^	0.331	−0.044	−0.084
**CNS** ^f^	−1.397	−1.289	−0.661
**CYP1A2** ^g^	Yes	Yes	Yes
**CYP2C19** ^h^	Yes	No	Yes
**ToPoSA (Å^2^)** ^i^	32.67	21.59	39.72
**Log P** ^j^	2.97	6.75	5.56
**B. S** ^k^	0.55	0.55	0.55
**LD_50_** ^l^	2.404	2.852	2.872
**HpT** ^m^	No	Yes	No
**AT** ^n^	No	No	Yes
**MToD** ^o^	0.066	0.446	2.258
**Lp. violation** ^p^	0	2	1
**ToC** ^q^	0.294	0.447	0.996

^a^ Molecular weight (Mwe).

^b^ Hydrogen bond acceptor (HBA).

^c^ Hydrogen bond donor (HBD).

^d^ Number of rotatable bonds (Num. rot.).

^e^ Blood‐brain barrier permeability (BBB).

^f^ Central nervous system permeability (CNS).

^g^ Cytochrome P450 (CYP) enzyme (CYP1A2).

^h^ Cytochrome P450 (CYP) enzyme (CYP2C19).

^i^ Topological polar surface area (ToPoSA).

^j^ Predicted octanol/water partition coefficient (Log P).

^k^ Bioavailability score (B.S.).

^l^ Oral rat acute toxicity (LD_50_).

^m^ Hepatotoxicity (HpT).

^n^ AMES toxicity (AT).

^o^ Maximum tolerated dose for human (MToD).

^p^ Lipinski violation (Lp. violation).

^q^ Total clearance (ToC).

#### 2.5.2 Examination of the Phyto‐Compounds' Thermodynamic Characteristics

To investigate how modifications to general parental compounds influence medicinal attributes (electronic power, Gibbs free energy, and enthalpy), Gaussian 6.0.16 was utilized to analyze overall dipole moment, charge distribution, as well as thermodynamic. Gaussian probabilities were used to compute its stoichiometry, which provided data regarding the chemical composition of the main medication along with its equivalent corresponding substitutes. Most of the remaining intermediates exhibit noteworthy thermo characteristics when compared to Diazepam. These results demonstrated how these various medications' predicted dipole moments were significantly changed. Their moment of dipole rises to strengthen whole polarization, hydrogen bonds, and nonbond connections between its drug‐receptor complexes. Predicting these encounters now heavily relies on bonding association‐free energies. Dynamic interaction along with collisions requires a negative free energy level, even if an optimistic free energy value illustrates that bonding will stay mostly unchanged. Intrinsic energy in all materials is negative. The aforementioned also relates to generic thermodynamic properties, thereby giving us the knowledge required to recognize the fundamental properties of macromolecules. Table 6, indicates that molecules along with the ones they were referring to possessed negative electrical strengths, which have the least negative energies and are necessary for immediate bonding to happen ‐1262.205 Hartree then Aniline has ‐1400.866 Hartree and Isoquinoline possesses the most negative energy ‐1479.829 Hartree, that showed noteworthy associations during interacting into 6CDU. It is also necessary for the antagonists to become more polarized as the dipole potential rises. Despite isoquinoline having the largest dipole potential (3.030 Debye), Diazepam has (2.889 Debye) and Aniline possesses the smallest dipole moment (2.864 Debye), along with its enthalpy and electrostatic conductivity.

**TABLE 6:** Investigation of thermodynamic and electronic aspects of diazepam, aniline, and isoquinoline.

Name	Stoichiometry	Electronic energy (Hartree)	Enthalpy (Hartree)	Gibbs free energy (Hartree)	Dipole moment (Debye)
**Diazepam (Control)**	C_16_H_13_ClN_20_	−1262.205	−1262.204	−1262.266	2.889
**Aniline**	C_33_H_23_NO	−1400.866	−1400.865	−1400.952	2.864
**Isoquinoline**	C_31_H_31_NO_3_	−1479.829	−1479.828	−1479.927	3.030

#### 2.5.3 Frontier Molecular Orbital (FMO) Analysis of the Phyto‐Compounds

Frontier orbits (FO) are made up of the topmost controlled molecular orbital (HOMO) and the bottom unoccupied molecular orbital (LUMO). In the process of determining the concentration of an expected medication which binds to a particular recipient, frontier orbital metabolic activities were employed to quantify chemical sensitivities. Conversely, HOMO might play a key role in creating a link between the overall technological parameters and how well healthcare equipment works. A greater dynamical consistency and limited chemical susceptibility are linked to a lengthier FO distance since it is often very undesirable to allow certain electrons to traverse across such low‐energy HOMO to reach a higher‐altitude LUMO. Frontier HOMO and LUMO frequencies were employed to evaluate both the softness as well as toughness of each substance's molecules. The accompanying formula was employed to ascertain the medications' hardness (*η*) as well as softness (*S*) utilizing the Parr and Pearson understanding of DFT alongside Koopmans theorem.

$$\eta =\left[\varepsilon \mathrm{LUMO}-\varepsilon \mathrm{HOMO}\right]/2$$

s=1/η

The whole chemical permanence was determined by this molecule's motion. To compute kinetic instabilities as well as chemical responsiveness, the rate of variability of HOMO along with LUMO. The result shows that the whole dynamic longevity increases as whole Hardness increases, even when overall variation as well as distances improve in parallel increasing of HOMO‐LUMO. Although significant chemical compassion, the differential contracts while respective softening indices increase, leading to reduced mechanical endurance. Softness (*S*) mark was to be found 11.904 for Diazepam, 11.765 for Aniline. The highest softness (S) degree was measured 9.900 for Isoquinoline, among all derivatives. It becomes clear that Isoquinoline possesses the chemical adaptability to interact with a particular chimeric human alpha1GABA_A_ receptor. Table 7 and Figure 4, show the HOMO‐LUMO, Softness and Hardness studies of ligands.

**TABLE 7:** HOMO‐LUMO, softness and hardness studies of ligands.

Molecules (chair)	εHOMO^a^	εLUMO^b^	Gap^c^	*Η* (Hardness, Gap/2)^d^	*S* (Softness, 1/Hardness)^e^
**Diazepam (Control)**	−0.215	−0.047	0.168	0.084	11.904
**Aniline**	−0.232	−0.061	0.171	0.085	11.765
**Isoquinoline**	−0.205	−0.002	0.203	0.101	9.900

^a^ Higher occupied molecular orbital (εHOMO).

^b^ Lower unoccupied molecular orbital (εLUMO).

^c^ Difference between HOMO and LUMO (Gap).

^d^ Hardness of the compound (*H*).

^e^ Softness of the compound (*S*).

**FIGURE 4:**
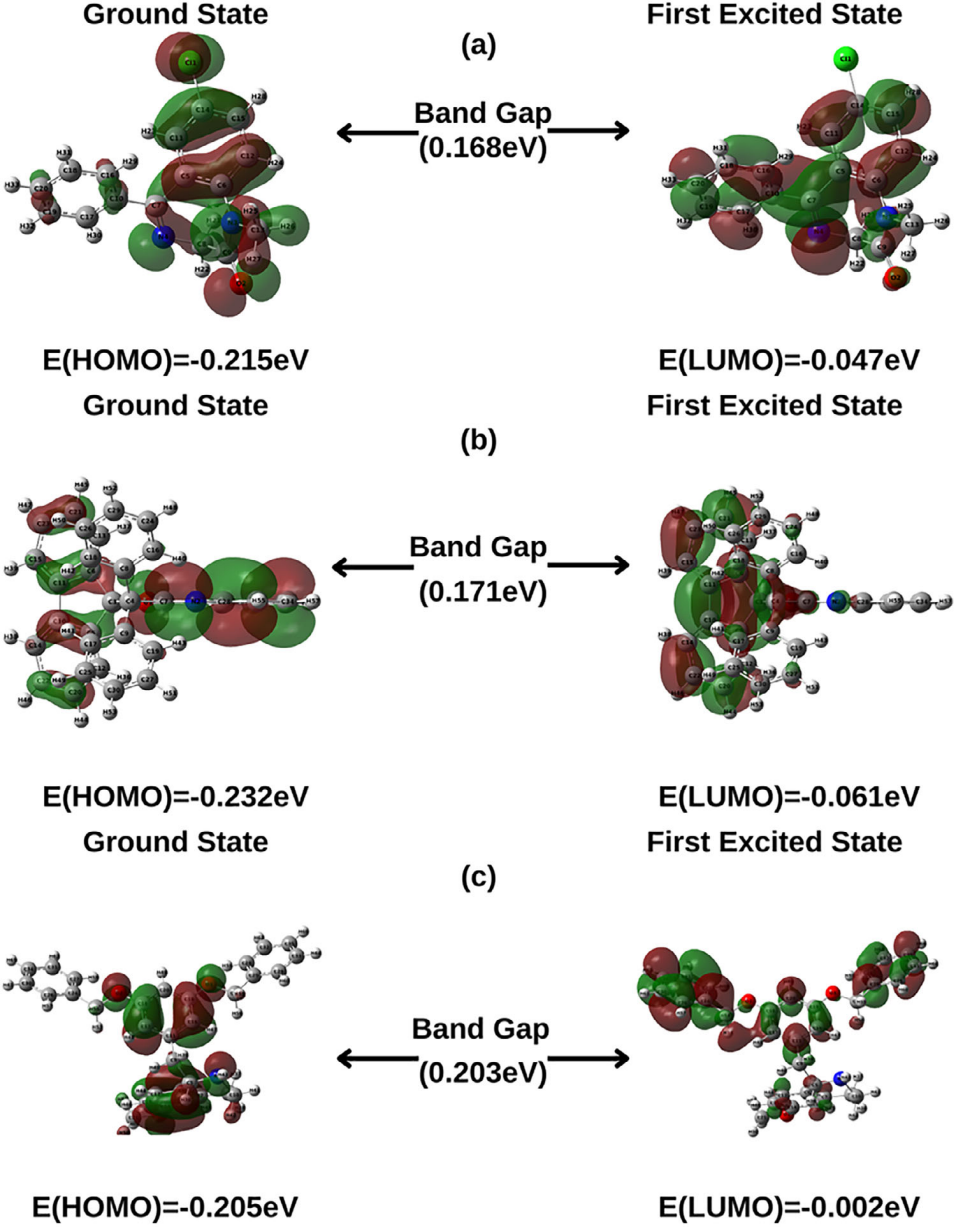
At the DFT/SDD level of theory in the gaseous state, plots of HOMO and LUMO molecular orbital distributions in the ground state of (a) Diazepam (b) Aniline (c) Isoquinoline.

#### 2.5.4 Site Specific Molecular Docking

By applying a molecular docking strategy utilizing the Maestro (Schrödinger Release 2021‐2: Maestro, Schrödinger, LLC, New York, NY, USA, 2020‐3), we were able to identify three selected phytocompounds bind effectively to the protein of interest chimeric human alpha1GABA_A_ receptor (PDB ID:6CDU). The maximum affinity for binding was obtained through the Maestro treatment for the entire ligand's assortment. The grid value was (X: ‐30.504, Y:49.201, and Z:7.553). The molecular binding attraction of Isoquinoline (CID‐595533) was −8.4 Kcal/mol. In contrast, the binding affinities of Aniline (CID‐630331) along with Diazepam (CID‐3016) were both lower than that of Isoquinoline, at −8.2 and −7.1 Kcal/mol, respectively. Table 8, represents the molecular interactions between the specified protein complex and the amino acid residues of the chosen phyto chemicals

**TABLE 8:** The molecular interactions between the specified protein complex and the amino acid residues of the chosen phyto chemicals are tabulated in the binding affinities scores.

Ligand–protein interaction	Bonding energy (Kcal/ mol)	H‐bond residues	H‐bond length (Å)	Number of H‐bonds	Other bond residues
**Diazepam (Control)+6CDU**	−7.1	Lys222	2.601	1	Glu159, Tyr225.
**Aniline+6CDU**	−8.2	—	—	0	Val410, Trp288, Phe407, Val292.
**Isoquinoline+6CDU**	−8.4	Lys222, Arg199	2.555, 2.952, 3.13	3	Glu159, Tyr225, Phe226, Ile228.

#### 2.5.5 Post Docking Protein–Ligands Interactions Analysis

A virtual examination technique along with the tools BIOVIA Discovery Studio as well as Ligplot+ V 2.2 were utilized during in silico study to find some of the best‐known confirmed drug‐ligand associations. When it came to docking values, Diazepam exhibited the least one (‐7.1 Kcal/mol), forming a single hydrogen bond with Lys222 and engaging in amino acid residues along with Tyr225 and Glu159 are connected by hydrophobic bonds. The binding strength of Aniline was −8.2 kcal/mol. It did not form hydrogen bonds through macro molecules. and instead, bonded hydrophobically to the remaining protein residuals, Trp288, Phe407, and Val 292. Out of all three possible drugs, Isoquinoline exhibited the greatest binding energies (−8.4 Kcal/mol). It forms three hydrogen interactions that attach to the macromolecule GABA_A_ through its Lys222 and Arg199 residues. It additionally possesses hydrophobic interactions between the other protein residuals, Glu159, Tyr225, Phe226, as well as Ile228. Figure 5, showing the post docking analysis of Diazepam, Aniline, and Isoquinoline respectively.

**FIGURE 5:**
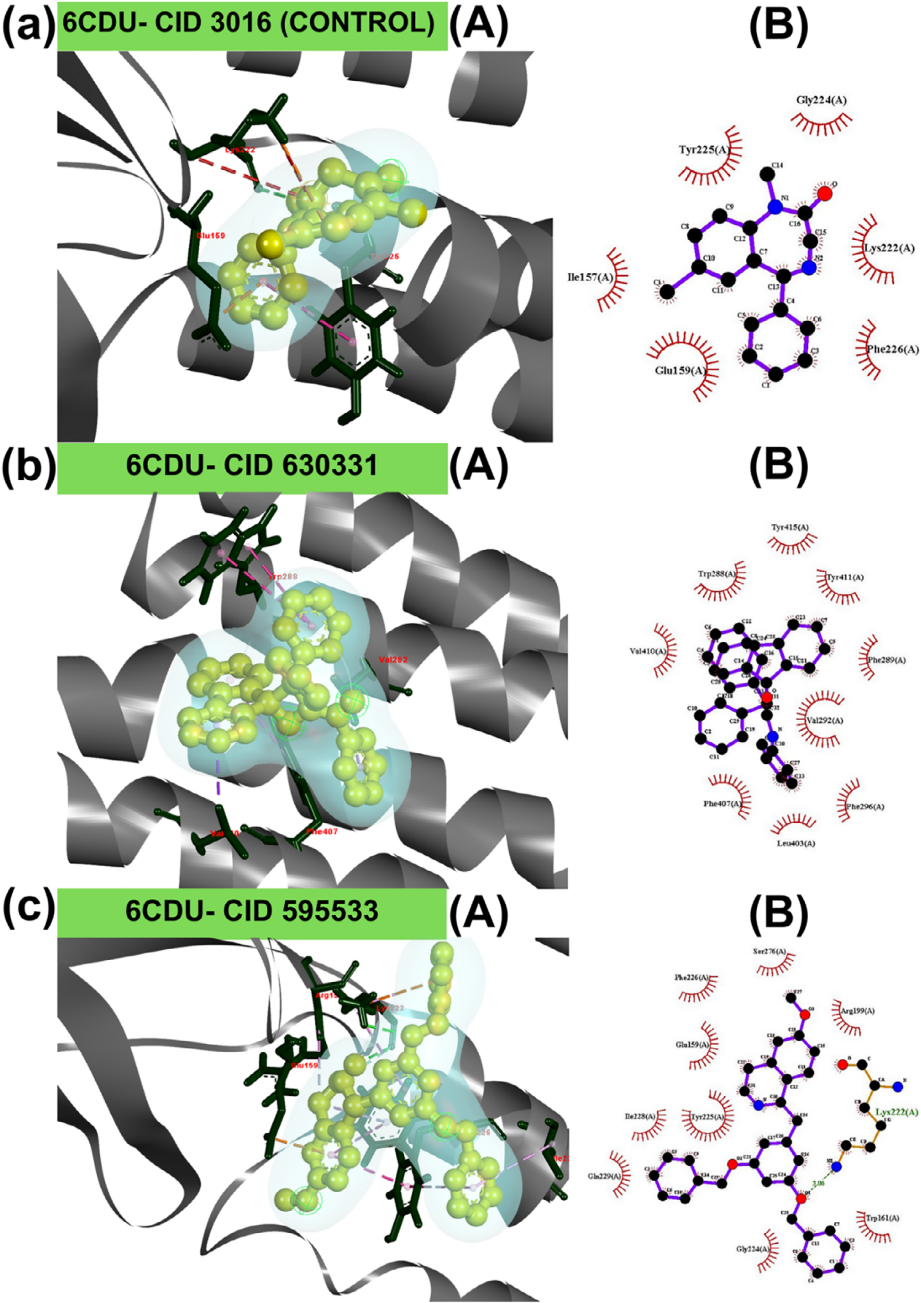
Post Docking Protein–Ligand interactions of (a) Diazepam, (b) Aniline, and (c) Isoquinoline.

### 2.6 Examination of MDS Trajectories

In computer‐aided drug development, MD simulation was employed to examine the stabilization and correspondence between molecules of an actual protein–ligand combination. Whenever an intricate mechanism is exposed to a simulated setting, such approach may additionally detect the conformational change. MD modeling of the protein's structure in respect to the particular ligands was done in this work in an effort to better understand the complex protein's structural changes over a 100 ns period. The terminal snapshots of 100 ns when analyzing intermolecular movement MDS progressions had initially been utilized.

#### 2.6.1 RMSD Analysis

The root means square deviation (RMSD) dimension was used to estimate the average variation across the displacement of a selected atom throughout a definite duration relative to a benchmark instant. The protein–ligand interaction's average shift in RMSD fell within an interval of 1 to 5, which was quite reasonable. Whenever the RMSD value exceeds 1–5, it shows that the protein composition has undergone substantial alteration. As a result, an MD simulation spanning 100 runs as well as the corresponding RMSD measurement was obtained to evaluate the alignment‐based modification of the required protein in a combination involving the two ligand molecules provided by Diazepam, Aniline, and Isoquinoline.

The main RMSD value of Diazepam ligand molecule is 1.0–1.2, which suggests less variations. The RMSD of ligands molecules of Isoquinoline ranged from 0.3 to 3.1 while Aniline was found to be between 2.1 and 2.8. The significance of chemical Diazepam fluctuated very little, which was not as great as the permitted range 2.0–2.1 showing the protein–ligand complex's structure's dynamic flexibility displayed in Figure 6A.

**FIGURE 6:**
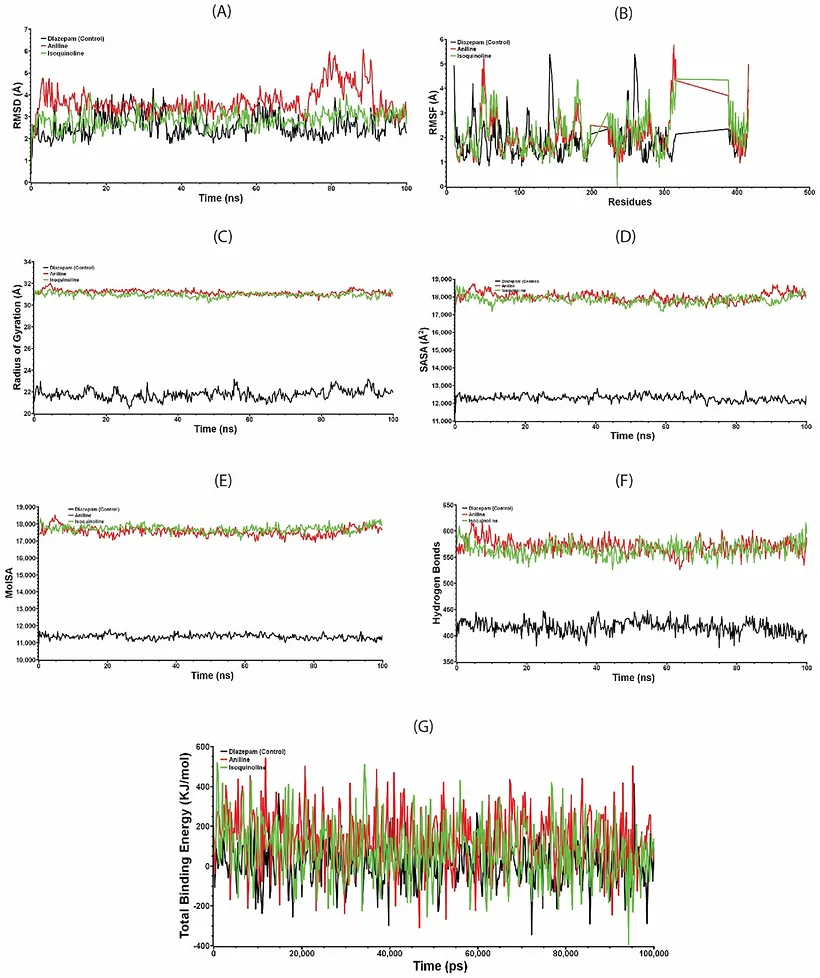
Molecular dynamics (100 ns) analysis of the target protein in complex with diazepam (control), aniline, and isoquinoline. Structural stability and flexibility were assessed by backbone RMSD (A) and residue‐wise RMSF (B), while compactness and solvent exposure were evaluated using radius of gyration (C), SASA (D), and MolSA (E). Intermolecular stability was further examined through hydrogen bond analysis (F) and total binding energy profiles (G), providing a comprehensive comparison of ligand‐induced dynamic behavior.

#### 2.6.2 RMSF Analysis

When chemicals bind to specific sections of proteins, quantitative root mean square fluctuation (RMSF) might be used to identify and describe distinct regional modifications happening between the chains of polypeptides. The localized changes that occur across the chain of proteins whenever specific ligands particles attach certain receptors may be better understood and identified using RMSF. As a result, the RMSF values of the compounds Diazepam, Aniline along with Isoquinoline using the intended protein model were determined to examine the alteration regarding the molecular elasticity of peptides resulting from attaching the selected ligand molecules to a specific residual site, as shown in Figure 6B.

Exceptionally stiff secondary structures, including beta‐ and alpha‐strands, were determined to gain a baseline observational rate ranging from 3 to 420 residues of amino acids. Most of the disparity was noticed at the beginning and end of the protein due to the N‐ and C‐terminal regions being present. As a consequence, it is possible to conclude that in the simulated setting, the moving of a single atom possessed a small likelihood of variation within the three binding molecules that were investigated, particularly Isoquinoline.

#### 2.6.3 *R*_g_ Analysis

The radius of gyration (*R*_g_) is sometimes defined as the atomic distribution throughout the shafts within a protein–ligand interaction substrate. Among the most crucial indicators, both illustrating modifications to overall complex compactness and predicting the actual structural behavior of a macromolecule is the computation of *R*_g_. The mechanism of protein–ligand engagement *R*_g_ may be defined as its atoms' orientation across its longitudinal axis. The measurement of *R*_g_ is one of the most vital indicators to look for when predicting the structural function of a macromolecule since it shows how changes in compactness get more complex with time.

As a consequence, as shown in Figure 6C, the stability of Diazepam, Aniline, and Isoquinoline while interacting with the protein of interest had been investigated during a 100 ns simulation period, with respect to *R*_g_. The mean *R*_g_ values pertaining to the substances Diazepam, Aniline, and Isoquinoline were determined to be 22.2, 35.5, and 32.8 correspondingly, showing that the protein's binding location did not undergo substantial conformational alterations as a result of engaging the whole chemicals.

#### 2.6.4 Analysis of SASA, MolSA, and IntraH

The development and operation of biological biomolecules are affected by the surface area that solvents may occupy (SASA). The outermost layers of peptides with unique amino acid sequencing function as activation regions most of the time. They might or might not incorporate with other elements such as receptors, that help in comprehending the solvent‐like characteristics (hydrophilic or hydrophobic) of a molecular structure in addition to protein–ligand combinations. The quantity of SASA regulates the molecular structure confirmation as well as the functioning of biological macromolecules. For the majority of instances, Proteins sequences that are amino acids function through active regions on their surface including/or engage different molecules and ligands, which increases comprehension of a molecule's solvent‐like characteristics (hydrophilic or hydrophobic) in accordance with the protein–ligand interaction components.

Consequently, the SASA measurement for the protein's complexes including the three ligands’ molecules was determined and represented in Figure 6D. The SASA measurements found for the substances Diazepam, Aniline and Isoquinoline ranged from 11200 to 18600 Å^2^, suggesting an elevated degree to which an amino acid's residue was exposed to the designated molecule in complicated systems.

The MolSA may be compared to a van der Waals surface area calculated alongside a probe diameter of 1.4 Å. Diazepam, Aniline, and Isoquinoline having the usual van der Waals surface area inside our computational simulation Figure 6E. Moreover, IntraH has a role in molecules’ function. Diazepam, Aniline, and Isoquinoline both showed elevated IntraH levels when paired with the intended protein Figure 6F.

#### 2.6.5 MM/PBSA

Positive energies determined model‐favorable binding, whereas the docked complexes' bindings free energies were obtained using the MM‐PBSA. A better and more efficient contact relationship between the macromolecule and phytochemical was shown by the docked combinations' comparatively constant interaction score in Figure 6G.

Initially, Diazepam's (Control) value was high. Afterward, it dropped sharply and continued to oscillate over time. On the other hand, Aniline, and Isoquinoline showed more distinct trend. The binding energy of Aniline was relatively stable but exhibited a steady increase, suggesting decreasing stability over time. Meanwhile, Isoquinoline showed moderate stability, rapid increase in binding energy. Additionally, it showed a higher pick significantly than the other two compounds, suggesting a reduced binding affinity as time progressed.

#### 2.6.6 PCA Analysis

The structural dynamics of Diazepam (Control), Aniline as well as Isoquinoline were examined using principal component analysis (PCA) and the result showed clear divergences. With data point widely dispersed along the primary variability axis (PCA 1 at 63.9%), Diazepam had the greatest degree of flexibility, indicating that it might readily change form. Its moderate dispersion along (PCA 2 at 17%) indicated that, although it had a lot of movement along with its primary flexibility axis, it was less flexible in other directions. Aniline had a narrower and more centered distribution (PCA 1 at 67.1%) and was somewhat more controlled, striking a balance between stability and flexibility. Its (PCA 2 at 17%) value was the same as that of the Diazepam, indicating comparable secondary variability. With the densely packed points (PCA 1 at 67.8%) along with (PCA 2 at 16.8%), Isoquinoline was the stiffest, suggesting a less dynamic, stable structure. Dihedral angles play a crucial role in all compounds, demonstrating how structural alterations were determined by variations in rotational motions. PCA analysis of Diazepam (Control), Aniline, and Isoquinoline was shown in Figure 7.

**FIGURE 7:**
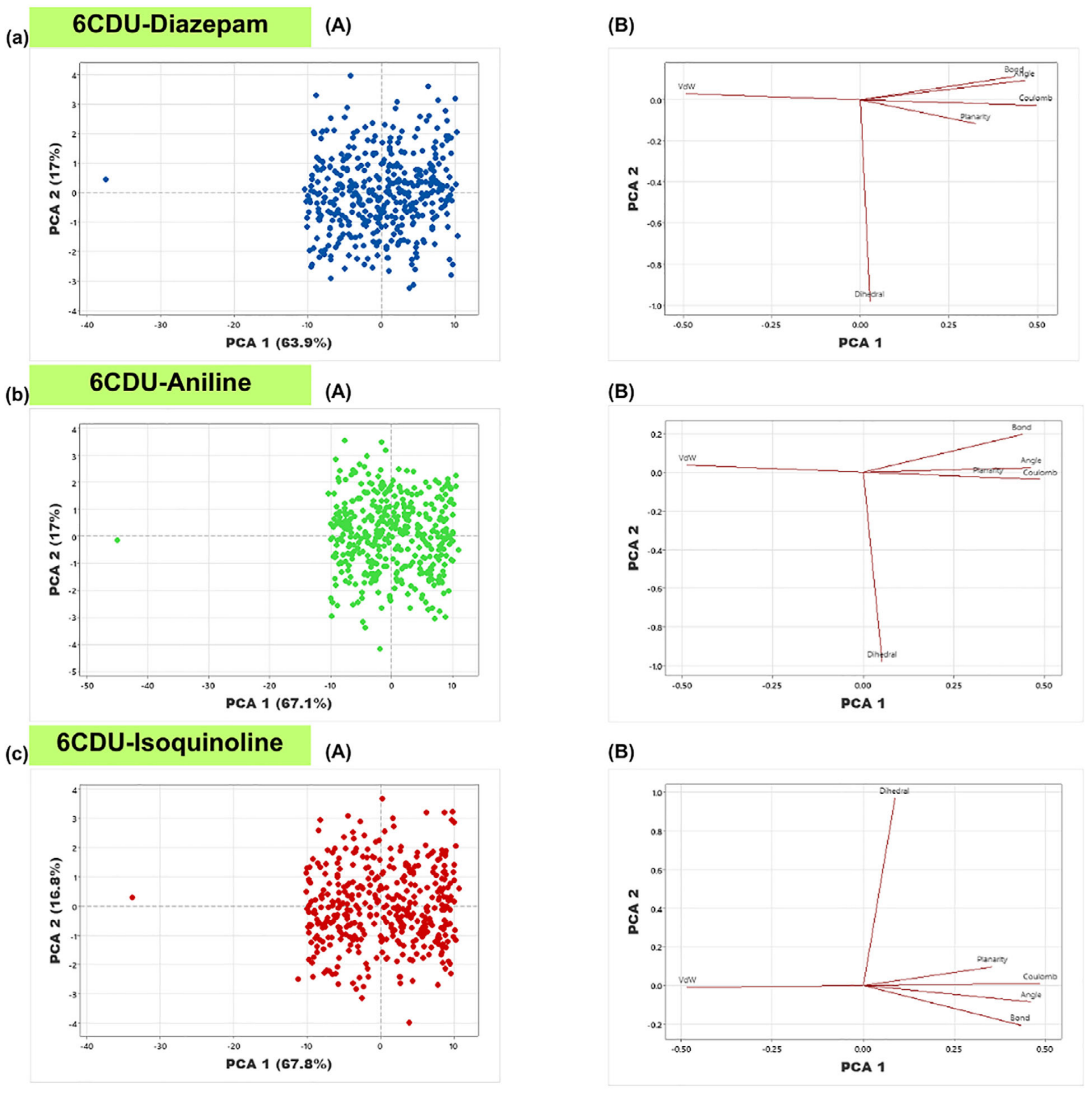
Principal component analysis (PCA) of (a) Diazepam, (b) Aniline, and (c) Isoquinoline descripting scores plots (A) for data distribution and loading plots (B) for description contributions (Bond, Angle, Dihedral, Coulomb, VdW) to PCA components.

## 3 Discussion

Anxiety is a pathological condition typically characterized by cynical feeling or sense of apprehension. From the evolutionary point of view, physiological anxiety plays vital role in the survival through anticipating to the external challenges and conserve self‐defense. Anxiety transforms from adaptive response to physiological manifestations which impair the quality of life [15].

Stress is well recognized triggering factor in the onset and development of anxiety disorder [16]. Chronic anxiety disrupts physiological homeostasis resulting series of biological malfunctions lead to ultimate deleterious health effects [17]. Oxidative stress, dysregulation of hypothalamic‐pituitary‐adrenal (HPA) axis and diminished neurotropic supports are central to the pathophysiology of anxiety [18]. Persistent circulation of high glucocorticoids, exclusively cortisol, is associated with anxiety‐related disorders [19].

Gamma‐aminobutyric acid (GABA), the most potent inhibitory neurotransmitter, plays central role in modulating anxiety behavior [20]. Thus, GABAergic has been vastly studied and targeted for the pharmacological management of neurological especially anxiety disorders. Several anxiolytics also work through GABA_A mediated pathways and attract attention of the scientists for future drug discovery [21, 22]. Additionally, serotonergic (5‐hydroxitriptamine) system play substantial role in regulating mood and emotional state. Therefore, lead(s) that modulate serotonergic system effectively control anxiety and related psychiatric disorders [23].

Consequently, GABAergic and serotonergic systems are predominant in anxiety‐like disorders and most of the researches focused on targeting these systems in developing pharmacological ailments [24].

The selective phytoconstituents found in SNE revealed excellent binding with GABAergic system responsible for anxiolytic effect through 6CDU (chimeric alpha1GABA_A_ receptors), which have been frequently employed in animal models of anxiety.

In the central nervous system, GABA is the primary inhibiting neurotransmitter [25]. The HBT is a recognized method for evaluating potential sedative and anxiolytic effects of any medication by monitoring the exploratory behavior of mice [26]. The methodological simplicity of this experiment is advantageous, as numerous behavioral reactions of an animal may be easily identified and quantified when introduced to a novel environment. It was discovered that the animals' head‐dipping habit is strongly tied to their mental condition. Based on this finding, it was hypothesized that a rise in head‐dipping actions might indicate the expression of anxiety in animals, whereas a decrease in the quantity of head dips may be connected to the sedative action. The use of EPMT test is considered to be better examining anxiety‐like behavior [27]. Anxiolytics reduce both the quantities of entries and quantity of period spend into the open arm. The results of the study are appropriate with the typical anxiolytic effects which provide evidence‐based use of the leaf for neurological disorder. Different plant derived phytoconstituents have benzodiazepine‐like nature which is capable of treating these neurological diseases. Flavonoids and tannins containing plants have shown activity in various CNS diseases [28]. It is likely that the anxiety‐reducing behavior demonstrated by SNE might be caused by the phytochemicals' ability to attach to GABA_A_ receptor and form benzodiazepine's complex. However, more detailed pharmacological investigations are required to prove it. In conducting neurological behavior by HBT, we have found that both of the fractions of SNE have reduced the number of head‐dipping frequency which indicated good anxiolytic effect. In the EPMT test, the time spent in closed arm was increased while both the number of entries and time spent in the open arm indicating good anxiolytic effect. We have also tested some antioxidative tests like DPPH, ABTS, and FRAP which also showed better oxidization properties of SNE which provide evidence for the restoration of anxiety in vivo.

Anxiolytic and neurodegenerative disorders occur due to oxidative damage and dysregulations of neuroreceptors like GABA [29]. Oxidative stress has been linked to brain disorders and symptoms that resemble anxiety [30]. The plant extract showed strong antioxidant effects in DPPH, ABTS, and FRAP tests, which means it can normalize oxidative stress leading brain function. Antioxidant potential may help stabilize the brain's chemical balance and support the GABA pathways, which play a pivotal role in reducing anxiety. Compounds having antioxidants properties are able to revive biomolecular and cellular changes improving neurogenesis [31].

From study results, we can suggest that the leaf of SNE contains different types of bioactive compounds that are responsible for antioxidants and anxiolytic potentials which would be helpful in treating anxiety and depression.

From GC‐MS analysis we have found few promising leads such as Dibutyl Phthalate, Naphthalene, Aniline, N‐(3',3'‐diphenylspiro [fluorene‐9,2'‐oxetan]‐4'‐ylidene), Isoquinoline, 1‐[(3,5‐dibenzyloxy) benzyl]‐1,2,3,4‐tetrahydro‐6 methoxy and many others. However, previous study has demonstrated that exposure to the above three substances, particularly Dibutyl Phthalate, extensively affects neuropharmacological behavior in vivo [5]. Moreover, naphthalene derived quinoxalines showed anxiolytic activity. However, Aniline, N‐(3',3'‐diphenylspiro [fluorene‐9,2'‐oxetan]‐4'‐ylidene) and Isoquinoline, 1‐[(3,5‐dibenzyloxy) benzyl]‐1,2,3,4‐tetrahydro‐6‐methoxy both are alkaloid and it's known to all that, alkaloids have effect on neuropharmacological behavior that is, anxiolytic effect [32].

In recent years, in silico approaches have become valuable tools in the early stages of drug discovery and development [33]. Molecular docking can provide an initial assessment of the possible binding orientation and predicted interaction strength of candidate compounds within a target binding site [34]. In the present study, candidate compounds were initially screened using molecular docking, and compounds were selected according to their predicted binding interactions. Among the screened compounds, Aniline, N‐(3',3'‐diphenylspiro [fluorene‐9,2'‐oxetan]‐4'‐ylidene) and Isoquinoline, 1‐[(3,5‐dibenzyloxy)benzyl]‐1,2,3,4‐tetrahydro‐6‐methoxy, showed the strongest predicted binding scores of −8.2 and −8.4 kcal/mol, respectively. Significant hydrophobic interactions and hydrogen bonds were observed between the compounds and receptor. These findings suggest favorable predicted interactions within the investigated receptor binding region. However, docking scores are computational estimates and cannot establish experimentally determined binding affinity, receptor activation or inhibition, or anxiolytic and antidepressant activity [35].

The two selected compounds were subsequently subjected to molecular dynamics (MD) simulation to investigate the structural behavior of the protein–ligand complexes. A 100 ns MD simulation was performed to evaluate the stability and conformational behavior of the investigated systems. The binding locations of the targeted compounds with the receptor showed similar active binding sites. The reported mean RMSD values were below 3 nm, while RMSF analysis showed relatively small variations among the conformations. *R*_g_ analysis did not indicate significant conformational alterations. Together, these findings support the structural stability of the investigated systems under the applied simulation conditions. SASA, MolSA, and IntraH analyses provided additional information regarding the structural and molecular behavior of the investigated systems and further supported the stability of the investigated conformations. Nevertheless, these observations represent the behavior of the modeled complexes during the simulation and do not independently demonstrate pharmacological efficacy or receptor modulation.

The MM/PBSA analysis indicated that the interactions of the investigated compounds with target protein 6CDU (chimeric alpha1GABAA receptor) were comparatively consistent and stable within the computational model. These findings provide additional computational support for the observed protein–ligand interactions; however, MM/PBSA values are computational estimates and should not be considered experimentally measured binding free energies [36]. PCA analysis showed significant flexibility in the investigated systems, providing information about collective conformational motions during the simulation [37]. Such flexibility may contribute to accommodation within the binding environment, but PCA alone cannot demonstrate improved binding or pharmacological efficacy [38].

The docking and MD interaction analyses provide complementary information regarding the interactions responsible for maintaining the investigated compounds within the receptor binding region [39]. Docking demonstrated hydrogen‐bond and hydrophobic interactions, while MD simulation was used to assess the stability of the complexes over time [40]. Comparison of these interaction profiles can therefore help determine whether the predicted binding mode was maintained during dynamic simulation [41]. However, the contribution of individual amino‐acid residues should be interpreted only according to the observed docking and MD interaction data and should not be used to infer a definitive mechanism of anxiolytic or antidepressant action.

A brief in silico ADMET analysis was performed to obtain preliminary information regarding the physicochemical, pharmacokinetic, and toxicity‐related characteristics of the selected compounds, with diazepam as a reference. The molecular weights of Aniline and Isoquinoline were 449.54 and 465.58 g/mol, respectively, compared with 284.74 g/mol for diazepam. Their predicted BBB values were −0.044 and −0.084, while CNS values were −1.289 and −0.661, respectively. TopoPSA values were 21.59and 39.72 Å^2^, compared with 32.67 Å^2^ for diazepam, and predicted LogP values were 6.75, 5.56, and 2.97 for Aniline, Isoquinoline, and diazepam, respectively. Both compounds had a bioavailability score of 0.55. Aniline showed 2 Lipinski violations, whereas Isoquinoline showed 1, compared with 0 for diazepam. The predicted total clearance values were 0.447, 0.996, and 0.294 for Aniline, Isoquinoline, and diazepam, respectively. These findings provide preliminary information on their predicted ADME characteristics but do not substitute for experimental pharmacokinetic studies.

The toxicity‐related parameters included LD_50_, hepatotoxicity (HpT), AMES toxicity (AT), and maximum tolerated dose (MToD). The predicted LD_50_ values were 2.852 for Aniline and 2.872 for Isoquinoline, compared with 2.404 for diazepam. Aniline was predicted as hepatotoxic, whereas Isoquinoline and diazepam were predicted as nonhepatotoxic. AMES toxicity was predicted as negative for Aniline and positive for Isoquinoline, while diazepam was negative. The predicted MToD values were 0.446, 2.258, and 0.066 for Aniline, Isoquinoline, and diazepam, respectively. These results indicate differences in predicted toxicity‐related profiles, but they remain computational predictions requiring experimental validation.

DFT‐based quantum mechanical calculations were also performed to characterize the electronic properties of the investigated compounds [42]. HOMO–LUMO energy‐gap analysis was used to evaluate their electronic characteristics and predicted chemical reactivity [43]. Several chemicals showed HOMO‐LUMO gap energies greater than 2.00 eV, which within the present computational analysis indicated comparatively lower chemical reactivity. However, this parameter alone cannot establish biological activity or pharmacological efficacy.

We could hypothesize whether aniline, N‐(3',3'‐diphenylspiro [fluorene‐9,2'‐oxetan]‐4'‐ylidene) and isoquinoline, 1‐[(3,5‐dibenzyloxy) benzyl]‐1,2,3,4‐ tetrahydro‐6‐methoxy may disrupt the chimeric alpha1GABA_A_ receptor. However, further investigation is needed to confirm the effects of aniline, N‐(3',3'‐diphenylspiro [fluorene‐9,2'‐oxetan]‐4'‐ylidene) and isoquinoline, 1‐[(3,5‐dibenzyloxy) benzyl]‐1,2,3,4‐ tetrahydro‐6‐methoxy.

## 4 Conclusion

The study included chemical profiling, biological assays, and in silico assessment to investigate the therapeutic properties of *S. nobilis* leaves. GC‐MS analysis identified two potential leads demonstrating possible candidates for anxiolytic effects in silico in future drug discovery. Behavioral studies supported anxiolytic effects in vivo. Strong free radical scavenging ability was also validated by antioxidant assays such as DPPH, ABTS, and FRAP, indicating that the plant extract may help in combating oxidative stress, connected to anxiety and neurodegeneration.

Altogether, the study provides compelling results in searching leads for anxiolytic effects. Further studies are required to isolate potential compound(s) from the plant for the future drug discovery in the treatment of neurological disorders.

## 5 Experimental Section

### 5.1 Plant Collection and Identification

Flowering shrub species *S. nobilis* was collected from subareas of Sundarbans, Khulna, and Bangladesh. We have paid special attention to eliminate any kind of counterfeit, and solely clean specimens of plants were used for the study. The plant material was identified by the expert taxonomist of the National Herbarium in Mirpur, Dhaka, and Bangladesh and voucher specimen with the accession number DACB 46857 was deposited for further documentation [5].

### 5.2 Extraction

The collected plant samples were shade‐dried to ensure the active constituents were free from decomposition and pulverized with a grinder to get powdered material. For the preparation of crude extract, 400 gm of *S. nobilis* powder was transferred to clean glass container, immersed in 2000 mL of 96% ethanol, with occasional shaking and mixing for 14 days. After filtration of the mixture, filtrate was evaporated using rotary evaporator (Model: SMART H150‐1000, Lab Tech), and obtained 21.98 g (yield = 5.495%) gummy crude *S. nobilis* extract (SNE).

### 5.3 Fractionation

We have used 18.395 g of crude leaf extract for fractionation. We have chosen solvents (*n*‐hexane, EtOAc and water) for fractionation based on polarity. The yield of *n*‐hexane, EtOAc and water fractions were 5.34 g (29.029%), 0.785 g (4.267%), and 9.86 g (53.601%) respectively.


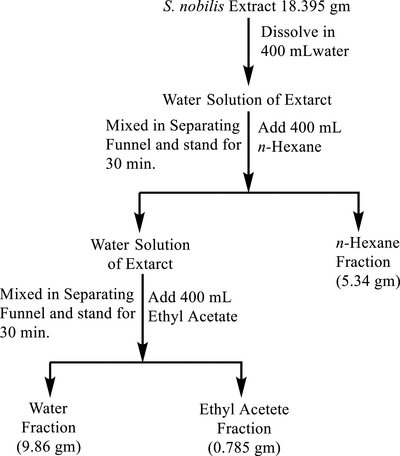


### 5.4 Animal

Young, male Swiss albino mice, 11–12 weeks old, with average weights of 25–35 g, were used in this study. The animals were acquired from Pharmacy Discipline, Khulna University animal house. We have maintained standard experimental condition for the mice for example, providing commercial rodent feed, water, photoperiodic regularity at 27 ± 2^°^C temperature. Animal experimental protocol was endorsed by the Animal Ethics Committee (AEC), Khulna University, Khulna, Bangladesh (Reference Number.: KUAEC‐2023‐05‐10). We have calculated the total number of mice for the experiments and the number of mice in each group using G*power analysis. We have distributed mice of in each group randomly to avoid study bias. We strictly complied ARRIVE 2.0 guidelines and 3Rs (Replacement, Reduction, and Refinement) principles to rationalize the use of animals.

### 5.5 Chemicals and Reagents

The common medication vincristine sulfate was collected from Beacon Pharmaceuticals Limited, Bangladesh, and ascorbic acid and diazepam were collected from Square Pharmaceuticals Limited, Bangladesh as a kind gift. DPPH, ABTS, potassium ferricyanide, trichloroacetic acid, ferric chloride, and other lab chemicals were purchased from Sigma Aldrich, India.

### 5.6 Evaluation of Anxiolytic Activity

#### 5.6.1 Hole‐Board Test (HBT)

With a few minor adjustments, this test was conducted using the previous outlined methodology [44]. We have randomly selected mice into six groups (10 mice in each). Control group received vehicle only, while standard treatment group received diazepam (1 mg/kg), plant treatment groups received two oral doses (250 and 500 mg/kg) of nonpolar and polar fractions of SNE. After 30 min, every animal was placed separately in the middle of a hole‐board, provided 5 min to roam around the device. The aggregate number of head‐dips was calculated. The anxiolytic activity was evaluated in percentile of inhibition in number of head‐dipping [45].

#### 5.6.2 The Elevated Plus Maze Test

The test had been carried out as earlier protocols with minor modifications considering the local context [46]. We have randomly selected mice into six groups (10 mice in each group). Only vehicles were provided to the control group, while standard treatment group received diazepam (1 mg/kg), plant treatment groups received two oral doses (250 and 500 mg/kg) of nonpolar and polar fractions of SNE. Each mouse was kept in the middle of the maze and allowed free movement for 5 min. The frequency of entries and total duration of time spent in the arms (both close and open arms) were measured. An entry into an arm is noted when the mouse fully crossed the defined lines of the respective arm [47].

### 5.7 Brine Shrimp Lethality Bioassay

The ability of SNE to kill laboratory‐cultured *Artemia salina* nauplii was carried out as stated by the established methodology [48]. Shortly, brine shrimp eggs were laid in order to produce nauplii. Varying concentrations ranging between 0.5162 and 640 µg/mL of nonpolar and polar fractions of SNE as well as vincristine sulphate had been prepared using serial dilution. Ten alive nauplii in 5 mL of prepared solutions were kept for 24 h. Finally, the number of alive nauplii was counted, and the percent mortality was calculated. The test was performed in duplicate to avoid experimental errors.

### 5.8 Evaluation of Antioxidant Activity

#### 5.8.1 DPPH Assay

The test was carried out based on the analogous approach outlined by Aiyegoro and Okoh [49]. Briefly, we have prepared DPPH solution (0.1 mmol/L) and stored it in the dark. Further we have added 200 µL of DPPH solution to the samples and standard. The concoction was mixed well and kept for 30 min at room temperature. We measured reaction's absorption potential mixtures at 517 nm spectrophotometrically. We have employed ascorbic acid as a reference medication to compare the samples' outcomes. Finally, he following formula was used to get the percentage of inhibition‐

$$\begin{array}{ccc} \%\;\text{of}\;\;\text{inhibition} & = & \left[\left(\text{absorbance of control}\right.\right.\\ & & \left.\left.-\;\text{absorbance of sample}\right)/\text{control}\right]\times 100\% \end{array}$$

#### 5.8.2 ABTS Assay

The test was conducted using the procedure described in [50] with few modifications. We have prepared a reaction mixture containing ABTS (7 mM) and potassium persulfate (2.45 mM), and kept it in the dark for 16 h. The mixture further was diluted with PBS (50 mM). Different concentrations of the extract and standard were prepared, and 150 µL ABTS solution was incorporated in to 50 µL of samples (both extract and standard). We took the absorbance of the mixtures after 30 min at 734 nm. The following formula was applied to get the inhibition percentage‐

$$\begin{array}{ccc} \%\;\text{of}\;\;\text{inhibition} & = & \left[\left(\text{absorbance of control}\right.\right.\\ & & \left.\left.-\;\text{absorbance of sample}\right)/\text{control}\right]\times 100\% \end{array}$$

Results of the extracts was compared against ascorbic acid standard.

#### 5.8.3 FRAP Assay

FRAP assay is a well‐established method of measuring antioxidant property. We have adapted the method from the earlier method [51] with few modifications. Briefly, we have prepared different concentrations of the extract and standard, and 2.5 mL of PBS and potassium ferricyanide were added into it and incubated (50°C) for 20 min. After the addition of 2.5 mL trichloroacetic acid, 2.5 mL of sample was taken from the mixture and 2.5 mL distilled water containing 0.5 mL FeCl_3_ was added into it, and incubated for 30 min. Finally, we measured the absorbance of the mixture at 593 nm. We have compared the results of the extract with the ascorbic acid standard.

### 5.9 GC‐MS Analysis

GC‐MS is a reliable method for identifying the chemical compounds in ethanol extracts of medicinal plants [52]. It helps reveal the bioactive components that may be responsible for the plant's therapeutic effects, making it essential in validating traditional herbal medicine [53].

The Clarus 690 gas chromatograph (PerkinElmer, CA, MA, USA) was utilized for GC‐MS analysis. It was outfitted containing a Clarus SQ 8 C mass spectrophotometer (PerkinElmer, CA, MA, USA) as well as a column (Elite‐35, 30 m 0.25 mm; PerkinElmer, CA, MA, USA) with 0.25 m film. A spitless mode was utilized to inject a 1 µL sample, and a steady flow rate of 1 mL/min of pure helium (99.999%) was employed as the carrier gas for the 40 min run. At a high energy of 70 eV, the sample was examined in the electron ionization (EI) mode. Despite a constant intake temperature of 280°C, the temperature of the column oven was set at 60°C, then expanded at a 213 pace of 5°C per minute to 240°C, where it remained for 4 min. One second was the scan duration, and the mass range was 50–600 *m*/*z* [54]. By comparing the sample chemicals with the National Institute of Standards and Technology (NIST) database, compounds were determined and presented according to the previous methods [55, 56, 57].

### 5.10 In Silico Analysis

#### 5.10.1 Ligand Preparation

GC‐MS analysis was performed to identify lead compounds in SNE, we found 27 bioactive compounds, which were used for further experiments. The identified substances underwent a virtual screening in order to find plausible alternatives for their anxiolytic and depressive properties against the chimeric alpha1GABA_A_ receptors. The spatial data file (SDF) that includes the tertiary arrangement of the chosen possibly bioactive aromatic substances and the control medication was obtained through the PubChem databases (https://pubchem.ncbi.nlm.nih.gov/). The LigPrep program from Schrodinger freeware (https://www.schrodinger.com/products/maestro) was used to create the structure of each ligand. Using the OPLS3e force field and the Epik ionizer, the minimizing procedure was performed in a typical pH range ranging from 7.0 to (+/) 2.0, with an RMSD of 1.0 and a maximum number of 11 conformists for every configuration.

#### 5.10.2 Protein Preparation

Using the Protein Data Bank (www.rcsb.org), the 3D x‐ray crystallographic structure of the chimeric alpha1GABA_A_ receptor (PDBID: 6CDU) was determined [58]. Next, using the conjugate gradients as well as the steepest descent methods of Swiss‐PDB Viewer 4.10, the protein's free energy decreased [59]. Using this method, the energy from 6CDU was reduced to −31376.07 kj/mol, whereas calculations were conducted in vacuo using GROMOS 96 43B1 specifications set lacking an induced field. PyMOL DLP 3D was then utilized to eliminate molecules of water and extraneous sequences from these structures of crystals [60].

#### 5.10.3 Pharmacokinetic Analysis of Phyto‐Compounds

Utilizing ADMET to calculate the ADME variables, a number of pharmacokinetic factors for pharmacologically active particles, were anticipated. Every 27 binding agents are being entered through the Swiss ADME database (http://www.swiss adme.ch/). Thereafter, just three substituents among the 27 compounds were evaluated for cytotoxicity, given that they did not even violate the Lipinski principle. The negative consequences of various potential antagonisms were investigated utilizing web‐based technologies; see ‘pKCSM’ (http://biosig.unimelb.edu.au/pkcsm/ prediction). In the end, three substances were chosen to proceed with further computerized study.

#### 5.10.4 Analysis of Thermodynamic Attributes of the Major Phytoconstituents

We have examined important thermodynamic characteristics of the major phytoconstituents of the extract using Gaussian 6.0.16 and the B3LYP/6‐31G (d, p) [61] phase of density—functional (DFT). Power and structure estimation and geometrical modification of freshly generated compounds are the main considerations.

#### 5.10.5 Frontier Molecular Orbital (FMO) Examination of the Major Phytoconstituents

The FMO determines the reactivity index of the compounds from which the degree of correlations between HOMO and LUMO can be measured and thus, suggested the prospect of the lead(s) as future drug molecule(s). Sensitivity is affected by several factors including the energy gap between HOMO and LUMO (*E*g), chemical softness (*S*), which is the reciprocal of hardness, and molecules' electrostatic perspective. Chemical hardness (*η*) was calculated by splitting the overall distance by two (HOMO and LUMO). Various sensitivity indices have been created employing the following formulas:

$$E_{g}=E_{\mathrm{LUMO}}-E_{\mathrm{HOMO}}$$

$$IP=-E_{\mathrm{HOMO}}$$

$$EA=-E_{\mathrm{LUMO}}$$

$$\eta =\frac{\left(E_{\mathrm{LUMO}}-E_{\mathrm{HOMO}}\right)}{2}$$

$$\mu =\frac{\left(E_{\mathrm{LUMO}}+E_{\mathrm{HOMO}}\right)}{2}$$

$$S=\frac{1}{\eta }$$

#### 5.10.6 Targeted Protein Optimization and Receptor Grid Formation

An attempt was made to obtain the preliminary tertiary structures of the protein of interest in a three‐dimensional (3D) format by searching the RCSB protein data bank (PDB) [62]. The layout of the targeted protein (PDB ID: 6CDU) with a resolution of 3.45Å obtaining 314 amino acids (AA) residues and a molecular weight of 37.55 kDa served as a receptor in the docking process. Once the file was installed, the Proteins Synthesis Wizard 12.5 of the Maestro Application (Schrödinger 2020‐3 Schrödinger, LLC, New York, NY, USA, 2020) was utilized to prepare the protein to be used. The chosen factors included the assigned bond orders, the chemical compound database (CCD), the addition of hydrogens, the creation of bonds of disulfide, the formation of zero‐order connections to metals, including the use of Prime to finish any sides of sequences that are lacking. Together having the provided capping termini as well as the elimination of fluids greater than 5 from temperature categories, we additionally employed Epik (Schrodinger Release version 2020‐3) to produce heat states with a pH of 7.0 ± 2.0. At a pH of 7.0, the H‐bond was found in PROPKA. The degradation stopped at an RMSD of 0.30 by lowering the heavy atoms saturation using the use of both the refinement tabs as well as the OPLS3e force field. Subsequently, the receptor's grids were put together by emphasizing the amino acid's native ligands as the focal point for its binding site.

#### 5.10.7 Best Lead Compounds Evaluation Through Site Specific Molecular Docking

The XP program (extra precision) site‐specific super molecular docking was carried out using Maestro (Schrödinger Release 2021‐2: Maestro, Schrödinger, LLC, New York, NY, USA, 2020‐3). Following the conclusion of the XP (extra precision) molecular docking process, each protein–ligand intricate structure within PDB structure was obtained into the anchored post‐viewing record of the Schrödinger Suite Software (https://www.schrodinger.com/) to feed post‐docking visualization, analysis of nonbond interactions, alongside assessment of hydrophilic properties as well as biological activity.

#### 5.10.8 Post‐Docking Visualization

Once Schrödinger Release 2021‐2: Maestro, Schrödinger, LLC, New York, NY, 2020‐3's extra precision (XP) method for site‐specific molecular docking was finished, the entire PDB structures containing the targeting proteins, including a few chosen ligands combinations were obtained further post‐docking investigation. Utilizing Ligplot+ version 2.2, noncovalent interactions (polar as well as hydrophobic) amongst complexes of protein–ligand were studied [63]. This visualization software turned out particularly effective by the Java interface (Java SE Runtime Environment 8u271) which only permitted the concatenated PDB documents generated by the Maestro program. Furthermore, the compact nature of molecular attachment, polar along with non‐polar interactions linkages among combinations, as well as various pertinent features were confirmed using the 64‐bit version of Discovery Studio Visualizer [64].

### 5.11 Molecular Dynamics Simulation

The YASARA dynamics program was utilized to carry out the suggested molecular dynamics simulations investigation, with the AMBER14 force fields [65]. The substances had hydrogen‐bonding geometries initially that had been changed and cleaned up. The TIP3P resolution concept was tested in shaped like cube simulating modules with ordinary hexagonal settings. The preparations for the simulation's compartments included 310 K, pH 7.4, and 0.9% NaCl. During the earliest stage of energy minimization, the sharpest gradient approaches (5000 cycles) were applied. Using approximately 8.0 cut‐off dimension and the Particle Mesh Ewalds methods, the extensive electrostatic processes undertaken are approximated. A duration period of 2.0 fs was applied to the systems for simulation. The simulated parameters were captured for every 100 ps. A Berendsen thermostat and constant tension was accustomed to improve the calculations for 100 ns. These channels were used to evaluate the radius of gyration (*R*_g_), solvent‐accessible surface area (SASA), bonds of hydrogen (IntraH), root mean square differences (RMSD), root mean square fluctuations (RMSF), molecular surface area (MolSA), molecular Mechanics Poisson–Boltzmann Surface Area (MM/PBSA) and principal component analysis (PCA) analysis.

#### 5.11.1 RMSD Analysis

The estimated dispersion that a single atom might undergo throughout a particular period of time was measured by the root means square deviations, or RMSD. It was evaluated in relation to an alternate period called the benchmark period. Specified atomic nuclei which comprise proteins, such as the C backbone, side chains, and bulkier particles, were initially subjected to a calculation of their relative mean square deviation (RMSD). These results were then situated and assessed in connection within the reference period. Considering a basic MD simulation with regularity x, the approximate RMSD might be computed using the formula below (Equation 1).

(1) $${\text{RMSD}}_{x}=\sqrt{\frac{1}{N}}\sum_{i=1}^{N}{\left(r_{i}^{\prime }\left(t_{x}\right)-r_{i}\left(t_{\text{ref}}\right)\right)}^{2}$$

Whenever an integer was selected in systems X, it was added to the structure after the positioning of the conventional technique was superimposed. The total r' quantity indicated the location of the bits in mechanism X after the midpoint of the indicating mechanism had been properly superimposed.

#### 5.11.2 RMSF Analysis

The primary goal of the RMSF is to detect and monitor modifications that impact target transcriptional morphology within polypeptides within a specific area of the resulting protein mixture. For a given number of repeats, the continuous formula continuity (Equation 2) could be used to get the overall RMSF amounts for the MD simulations for a particular polypeptide.

(2) $${\text{RMSF}}_{i}=\sqrt{\frac{1}{T}}\sum_{t=1}^{T}\left\langle {\left(r_{i}^{\prime }\left(t\right)-r_{i}\left(t_{\text{ref}}\right)\right)}^{2}\right\rangle$$

In the majority of instances, the duration *T* represents progress, although in some instances, *T*_ref_ represents the starting point or supplied time. From these values, the diagonal measurements travelled through sequencing b which were aggregated as shown by (>), and r' shows where the selected atoms fit inside frames I after being transformed upon its conventional setting up.

### 5.12 Statistical Analysis

We have evaluated assumptions of parametric tests of our data. Normality test was calculated using the Shapiro–Wilk test. As the data sets satisfied parametric test, we have used one‐way ANOVA followed by multiple comparison by turkey post hoc analysis using GraphPad Prism software (version 21) (GraphPad Software, California, USA) (www.graphpad.com). The results of the experiment in this study are represented as mean ± standard mean of error (SEM). *p* ≤ 0.05 is considered to be the statistically significant data.

### 5.13 Limitation

We did use authentic standards to confirm the GC‐MS compounds. We have matched our compounds found in the GC‐MS with NIST library database. Brine shrimp lethality test was carried out to evaluate cytotoxicity. Future studies are required to confirm the cytotoxicity of the plant materials. We have speculated the leads as prospective potential anxiolytic candidates for future drug discovery based on molecular docking and dynamics simulation studies. Further studies are required to synthesize the leads and conduct more detail experiments to confirm its toxicological and therapeutic effects.

## Authors Contributions

**Progga Paramita Paul**, **Musfiqur Rahman Sakib**, **Dabashis Chakraborty**, **Tasnim Fatema,** and **Trina Mitra**: investigation, formal analysis, software, and validation. **Pritam Kundu**, **Md. Shahriar Hossen,** and **Md. Ahsan Abid**: data curation, supervision, visualization, and writing original draft. **Utpal Kumar Karmakar** and **Manik Chandra Shill**: conceptualization, funding acquisition, project administration, resources, and writing review and editing.

## Conflicts of Interest

The authors declare no conflicts of interest.

## AI Disclosures

Authors declare that they did not use any AI tools for drawing or writing the manuscript.

## Data Availability

All of the results presented here are original and authors will be fully responsible for any liabilities regarding this manuscript. All the experimental data are preserved by the authors. Those may be provided upon request.

## References

[1] F. Fontanella , T. D. Alessandro , E. Nardone , C. D. Stefano , C. Vicidomini , and G. N. Roviello , “Artificial Intelligence for Natural Products Drug Discovery in Neurodegenerative Therapies : A Review,” Biomolecules 16 (2026): 1–36. 10.3390/biom16010129 PMC12838884 41594669

[2] A. Licata , S. Ciriminna , O. Tulone , et al., “Herbal, Dietary Supplements, Oligo and Trace Elements Consumption Among Chronic Liver Disease Patients and Caregivers: A Cross‐sectional Study,” Internal and Emergency Medicine (2026): 1–9, 10.1007/s11739-026-04425-8.42334764

[3] Y. Paopun , P. Thanomchat , N. Kronburee , Y. Detpakdee , and S. Sukkhaeng , “Leaf Surface Microstructure, Anatomical Characteristics and Cystoliths of Phlogacanthus Pulcherrimus T. Anderson,” Chiang Mai Journal of Science 53, no. 3 (2026): 1–13, 10.12982/CMJS.2026.038.

[4] S. N. Wagh , A. Barkale , A. V. Kshirsagar , et al., “Herbal Abortifacients as Natural Alternatives to Synthetic Drugs: A Review on Efficacy, Safety, and Mechanisms,” Oriental Studies 26, no. 3 (2021): 17–144.

[5] P. P. Paul , P. Kundu , and U. K. Karmakar , “Chemical and Biological Investigation of *Sanchezia nobilis* Leaves Extract,” Jordan Journal of Pharmaceutical Sciences 15, no. 1 (2022): 121–131, 10.35516/jjps.v15i1.299.

[6] G. Koochakpoor , A. Salari‐moghaddam , and M. Bagheri , “Association Between Oxidative Balance Score and Psychological Disorders in Adults,” Scientific Reports 16 (2026): 18338. 10.1038/s41598-026-46301-7 PMC13263336 42286033

[7] T. Luo , “Suanzao Ren Decoction Alleviates Anxiety‐Like Behavior and Cognitive Dysfunction in Chronic Unpredictable Stress Rats by Modulating Oxidative Stress and Autophagy,” Brain and Behavior 16 (2026): 1–10, 10.1002/brb3.71585. PMC13366388 42444588

[8] N. M. Oruç , “Optimization of Solvent‐Based Extraction Conditions for Phytochemical Components From Laurus Nobilis L,” International Journal of Chemistry and Technology 10 (2026): 1–6.

[9] M. M. Shokr , A. M. Abu , E. Seham , and M. Al Raish , “Plant ‑ Derived Natural Products in Neurological and Psychiatric Disorders : Mechanisms of Action and Synergistic Roles With Probiotics,” Molecular Neurobiology 63 (2026): 766. 10.1007/s12035-026-06065-7 PMC13373017 42455433

[10] A. Gangwal and A. Lavecchia , “IMPACT Framework : Establishing Global Standards for Artificial Intelligence Implementation, Methodology, and Translation in Drug Discovery,” Wiley Interdisciplinary Reviews: Computational Molecular Science 16 (2026): e70072, 10.1002/wcms.70072.

[11] E. S. Ozdemir , H. Jang , O. Keskin , and A. Gursoy , “Deep Generative Molecular Design and Its Value in Modern Drug Discovery,” Expert Opinion on Drug Discovery 21, no. 3 (2026): 273–287, 10.1080/17460441.2026.2636192. 41778634

[12] S. Akhtar , V. Ahmad , M. Aatif , Q. Mohammad , and S. Jamal , “Effect of Intercropping Mungbean on Maize and Sunflower Under Weed‐free and Weedy Conditions,” Journal of King Saud University—Science 38, no. 4 (2026), 10.25259/JKSUS.

[13] S. Rosignoli , E. Lustrino , O. Shevchuk , et al., “Bioinformatics‐Driven, Plant‐Based Antibiotic Research against Quorum Sensing and Biofilm Formation in *Pseudomonas aeruginosa* and *Escherichia coli* Multiresistant Microbes,” Biomolecules 16 (2026): 1–40. 10.3390/biom16020197 PMC12938168 41750267

[14] A. Gangwal , “From Medicinal Plants : Current Applications and Infrastructural Challenges,” Chemical Biology & Drug Design 108 (2026): e70372, 10.1111/cbdd.70372. PMC13425709 42535626

[15] L. Liu , M. Huang , X. Liu , et al., “The Extra Burden : Differential Somatization‐ Moderated Mediation Models of Anxiety, Depression, and Insomnia on Quality of Life Through Abnormal Illness Behavior in Chinese College Students,” BMC Psychology 14 (2026): 970. 10.1186/s40359-026-04461-1 PMC13330162 42001197

[16] N. Peixoto and R. Fonseca , “Personalized Resilience : How Individual Variability in Brain—Immune Responses to Stress Influences the Development of Anxiety Disorders,” Frontiers in Cellular Neuroscience 20 (2026): 1745994, 10.3389/fncel.2026.1745994, March. PMC12991989 41853172

[17] Y. Tan , “A Review of Biological Pathways of Chronic Stress as a Risk Hub for Multiple Psychosomatic Diseases from the Perspective of Clinical Nursing,” Nursing Research and Practice 1 (2026): 9570388, 10.1155/nrp/9570388. PMC13199855 42200148

[18] W. Dhahbi , K. Chamari , I. Dergaa , et al., “A Tripartite Framework of Conflict‐Induced Stress, Acoustic Hypervigilance, and Ramadan Observance as Convergent Determinants of Circadian Decoherence in Athletes A Tripartite Framework of Conflict‐Induced Stress, Acoustic Hypervigilance, and Ramadan Observance as Convergent Determinants of Circadian Decoherence in Athletes,” Nature and Science of Sleep 1608 (2026): 621788, 10.2147/NSS.S621788. PMC13455029 42577699

[19] D. Borakowska , “Adaptive Changes in Adrenal Steroid Metabolism Under Extreme Physical and Psychological Stress: A Narrative Review,” Frontiers in Endocrinology 17 (2026): 1860059, 10.3389/fendo.2026.1860059. PMC13368683 42460312

[20] P. Udawant , D. Dube , P. Vadhane , and S. Patil , “Evaluation of the Anxiolytic Potential of 4‐ Hydroxypiperidine on Swiss Albino Mice Ecperimental Animals,” Genetics and Molecular Research 25 (2026): 1–12.

[21] M. Gałuszka , “The Use of Selected Medicinal Plants in the Regulation of the Autonomic Nervous System Through the Neurotransmitter System During Stress,” Journal of Education, Health and Sport 94 (2026): 72859–72859.

[22] L. Constância de Melo Alves Silva , L. Ludmyla Sousa de Farias , V. Raquel de Lima , et al., “Integrative and Complementary Practices in Intensive Care Units: An integrative review,” Heliyon 10, no. 22 (2024): e40333, 10.1016/j.heliyon.2024.e40333. PMC11616508 39634435

[23] S. Pardossi , D. Koukouna , A. Cuomo , M. Pinzi , G. Piumini , and A. Fagiolini , “Fibromyalgia as a Neuroimmune‐Psychiatric Disorder,” Italian Journal of Psychiatry 12 (2026): 1–8, 10.36180/2421-4469-2025-1390.

[24] L. Pretorius , T. Moodley , and C. Smith , “Sex‐Specific Neurological Dysregulation May Underpin Distinctly Different Male and Female Behavioural Phenotypes in a Zebrafish Model of Autism Spectrum Disorder,” Behavioral and Brain Functions: BBF 22, no. 44 (2026): 1–18, 10.1186/s12993-026-00353-x. PMC13617892 42402578

[25] B. Vaibhav and R. K. Roy , “Balancing Excitatory and Inhibitory Neurotransmission in Epilepsy : Therapeutic Role of Gabaergic and Glutamatergic Modulation—A Review,” World 5, no. 5 (2026): 771–779.

[26] A. Begum , et al., “Phytochemical and Pharmacological Potentials of Edible Lannea Coromandelica Fruit: A GC–MS Supported, In Vitro, In Vivo, and In Silico Studies,” Scientific Reports (2026), 10.1038/s41598-026-61492-9.

[27] Y. Feng and T. Pang , “Fecal Microbiota Transplantation from Patients with Social Anxiety Disorder Is Associated with General Anxiety‐Like Behavior and Gut Microbiota Alterations in Mice,” Brain and Behavior 16 (2025): 1–11, 10.1002/brb3.71561. PMC13272884 42304699

[28] A. Sharma and S. Kaur , “Flame of the Forest: A Review on the Traditional and Modern Uses of Butea Monosperma,” Current Pharmaceutical Research 2, no. 2 (2026): 205–217, 10.63785/cpr.2026.2.2.205217.

[29] D. Avni , O. Weissberg , N. Pintel , and L. Izraelov , “Healing From the Ocean: Targeting Shared Mechanisms in Autism and Epilepsy Using Algae‐Derived Compounds,” Marine Drugs 24 (2026): 1–48. 10.3390/md24080277 PMC13514737 42646477

[30] P. Alam , S. Arabia , S. Kumar Jain , G. G. Vishwavidyalaya , P. Mishra , and S. B. Singh , Natural Products and Herbal Strategies in Covid‐ 19 and Mental Health Management Editors, (Mantra Publication, 2026).

[31] L. Shi , J. Wei , R. Chen , and Y. Liu , “From Mitochondrial Oxidative Stress to Neuroin Fl Ammation : Integrated Proteomic and Transcriptomic Pro Fi Ling Reveals the Role of the ROS /TXNIP / NLRP3 Signaling Pathway in Anxious Depression,” Frontiers in Immunology 17 (2026): 1803956, 10.3389/fimmu.2026.1803956. PMC13226016 42238570

[32] S. Mansur , T. Ahmed , A. Hakim , et al., “Macaranga Peltata Alleviates Neuropsychiatric Disorders in Mouse Model and Computational Study,” Food Science & Nutrition 14 (2026): 1–14, 10.1002/fsn3.71484. PMC12830868 41583015

[33] R. Chikhale , “The Expectations of in Silico Fragment‐based Drug Design and Future Challenges,” Expert Opinion on Drug Discovery 21, no. 2 (2026): 161–171, 10.1080/17460441.2026.2623154. 41630652

[34] A. Bisht , P. Sharma , S. Kumar , and V. Kumar , “In Silico Evaluation of Picroside I and Picroside II From Picrorhiza kurroa as Potential EGFR Tyrosine Kinase Inhibitors for Non‐small Cell Lung Cancer,” In Silico Research in Biomedicine 2, no. April (2026): 100472, 10.1016/j.insi.2026.100472.

[35] J. S. León‐magdaleno and J. M. Hernández‐meza , “In Silico Screening, ADMET Analysis, MD Simulations, and MM/PBSA Binding Free Energy Identify New Inhibitor Molecules for Viroporin E'' 7022,” Molecular Simulation 52 (2026): 618–645, 10.1080/08927022.2026.2659199.

[36] V. Kumari and S. Samanta , “Molecular Dynamics and Free Energy Analysis of Curcumin Binding Near the DNA‐Recognition Interface of NF‐κB,” Scientific Reports 16 (2026): 26447. 10.1038/s41598-026-56039-x PMC13500845 42270912

[37] J. Matematika , I. Alam , K. Angkasa , et al., “Penerapan Principal Component Analysis (PCA) Untuk Reduksi Dimensi Dan Pemetaan Karakteristik Nutrisi Pada Produk Makanan Kemasan Di Indonesia,” Dengan Memilih Makanan Kemasan Yang Lebih Sehat Dan Lebih Baik Sehingga Dapat Mencegah Makanan Kemasan Dan Tantangan Kesehatan Masyarakat 4 (2026): 20–30.

[38] M. Qureshi , M. Ismail , M. Daniyal , et al., “Characterizing Critical Sources of Carbon Emissions Using Principal Component Analysis,” The Scientific World Journal 1 (2026): 6175776, 10.1155/tswj/6175776. PMC13071754 41969135

[39] G. Padilla‐bernal and L. D. Herrera‐zúñiga , “Molecular Dynamics and Electron Density Topology Reveal Ligand‐Specific Interaction Patterns at the Dopamine D 2 Receptor,” International Journal of Molecular Sciences 27 (2026): 1–19. 10.3390/ijms27156572 PMC13465887 42589229

[40] S. E. Korcan , et al., “First‐Time Demonstration of a Two‐Step Mechanism for Azo Dye Removal by Bacillus Proteolyticus Using Molecular Docking and FT‐IR,” Water Environment Research 98, no. 8 (2026): e70524, 10.1002/wer.70524. PMC13454158 42572132

[41] B. Chen , G. Li , R. Liu , et al., “From Virtual Screening to Functional Validation : An MM/GBSA and Molecular Dynamics Driven Strategy for Targeting Phytopathogenic Fungal Ferrochelatase 1,” Journal of Integrative Agriculture (2026), 10.1016/j.jia.2026.06.031, n.d.

[42] S. Naciri , H. E. L. Ouafy , M. Boutkbout , et al., “Theoretical Analysis of a Pyrrole‐Based Compound : Insights Into Electronic Properties and Spectroscopic Behavior via DFT and TD‐DFT,” Chemical Review and Letters 9 (2026): 640–653.

[43] R. S. Silva , “Designing Diaryl Sulfide Electronic Properties: A Comprehensive DFT Guide to HOMO–LUMO Gaps and Reactivity Descriptors,” ACS Omega 11 (2026): 26328–26344, 10.1021/acsomega.5c11432. PMC13177040 42146242

[44] S. P. Aswathy , A. Jaison , S. A. Anjukrishna , and M. Manish Mohan , “Chronic Effects of Monosodium Glutamate on Anxiety and Motor Function in Swiss Albino Mice : Evaluation Using Hole Board and Rear Cylinder Tests,” International Journal of Pharmacy Research & Technology (IJPRT) 16, no. 1 (2026): 4961–4966, 10.31838/ijprt/16.01.687.

[45] T. Binte , B. Kabbo , S. Rana , et al., “Evaluation of Neuropharmacological Activities of Different Fractions of Annona Reticulata Linn. Leaves,” Biomedical and Pharmacology Journal 19, no. March (2026): 752–766.

[46] C. Eric , “The Elevated plus Maze for the Assessment of Activity and Anxiety in a Wild Subterranean Rodent : Role of Illumination and Mystacial Vibrissae,” Revista Argentina de Ciencias del Comportamiento (RACC) 18 (2026): 68–75.

[47] Z. Pei , D. Hu , J. Sun , and W. Hu , “Paternal Glufosinate Ammonium Exposure Leads to Memory Dysfunction in Offspring Mice,” Toxics 14 (2026): 1–16. 10.3390/toxics14050396 PMC13211454 42198522

[48] T. X. Bui , M. N. Tran , T. H. Tran , and T. T. H. Dang , “Flavonoids and the Subchronic Toxicity, Antipeptic Ulcer, and Analgesic Effects of the Ethyl Acetate Soluble Fraction of the Ethanol Extract From Sanchezia Nobilis Hook. F Leaves,” Natural Product Communications 17, no. 9 (2022): 1934578X221126306, 10.1177/1934578X221126306.

[49] S. mainoo , L. Tima , A. Acheampong , et al., “Pharmacological Properties of the Ethanolic and Petroleum Ether Extracts of the Stem of Salvia Splendens,” Journal of Medicinal Plants Research 19, no. 9 (2025): 176–186, 10.5897/JMPR2025.7402.

[50] P. Chaikham , P. Prangthip , A. Chotiko , W. Pathom‐aree , S. Rawdkuen , and S. Jirasatid , “Fermented Thai Sacred Lotus Stamens as a Source of Neuroprotective Phytochemicals: Changes in Bioactive Composition, Antioxidant Activity, and Alzheimer's Disease‐Related Enzyme Inhibition,” Food Chemistry Advances 12 (2026): 101382, 10.1016/j.focha.2026.101382.

[51] I. L. Lawag , S. Sultana , and L. Y. Lim , “Optimisation and Validation of a Microscale FRAP Assay With Matrix‐Matched (Interaction‐Corrected) Blanking for Accurate Antioxidant Capacity Assessment of Honeys,” Methods and Protocols 9 (2026): 1–33. 10.3390/mps9040120 PMC13515261 42646515

[52] O. Hope , I. E. Bright , and A. I. Alagbonsi , “GC‐MS Biocomponents Characterization and Antibacterial Potency of Ethanolic Crude Extracts of Camellia Sinensis,” SAGE Open Medicine 10 (2022): 20503121221116859, 10.1177/20503121221116859. PMC9373190 35966211

[53] R. Ramya and S. R. Dutta , “GC ‑ MS Analysis of Bioactive Compounds in Ethanolic Leaf,” Applied Biochemistry and Biotechnology 194 (2022): 600018. 10.1007/s12010-021-03742-2 34762268

[54] S. Triloknadh , C. Venkata , K. Nagaraju , et al., “Bioorganic & Medicinal Chemistry Letters Design, Synthesis, Neuroprotective, Antibacterial Activities and Docking Studies of Novel Thieno [2, 3‐ d] Pyrimidine‐Alkyne Mannich Base and Oxadiazole Hybrids,” Bioorganic & Medicinal Chemistry Letters 28 (2018): 1663–1669. 10.1016/j.bmcl.2018.03.030 29602681

[55] M. N. H. Zilani , M. A. Islam , P. Biswas , et al., “Metabolite Profiling, Anti‐Inflammatory, Analgesic Potentials of Edible Herb Colocasia Gigantea and Molecular Docking Study Against COX‐II Enzyme,” Journal of Ethnopharmacology 281, no. August (2021): 114577, 10.1016/j.jep.2021.114577. 34464698

[56] S. Rahman , N. Hasan Zilani , A. Islam , and M. Hasan , “In Vivo Neuropharmacological Potential of Gomphandra Tetrandra (wall.) sleumer and In Silico Study Against β‐Amyloid Precursor Protein,” Processes 9, no. 8 (2021): 1449.

[57] C. Sarkar , M. Mondal , K. Al‐Khafaji , et al., “GC–MS Analysis, and Evaluation of Protective Effect of Piper Chaba Stem Bark Against Paracetamol‐Induced Liver Damage in Sprague‐Dawley Rats: Possible Defensive Mechanism by Targeting CYP2E1 Enzyme Through in Silico Study,” Life Sciences 309, no. September (2022): 121044, 10.1016/j.lfs.2022.121044. 36208657

[58] B. Vallat , Y. Rose , D. W. Piehl , et al., “RCSB Protein Data Bank : Delivering Integrative Structures Alongside Experimental Structures and Computed Structure Models,” Nucleic Acids Research 54 (2026): 489–498, 10.1093/nar/gkaf1187. PMC12807596 41206752

[59] X. Wei , “AIQM‐PBSA : Integrating Machine Learning Interatomic Potentials With MMPBSA for Accurate Protein—Ligand Binding Free Energy Calculations,” The Journal of Physical Chemistry 130 (2026): 4982–4993. 10.1021/acs.jpcb.6c00306 42081673

[60] K. Gurning , Y. Steven Kurniawan , F. Septiani Silitonga , et al., “Analysis of Alkaloids Compounds and Antimalarial Activity of Coleus Amboinicus Leaves From Indonesia,” Trends in Sciences 23, no. 2 (2026): 1–16.

[61] A. Moin , Z. A. Asiri , M. A. Hamod , et al., “Scientific Reports Article in Press Computational Exploration of Anticancer Drug Adsorption on a Porous Organic Nanocage : Insights From Density Functional Theory for Targeted Nanocarrier Design,” Scientific Reports 16 (2026): 20646. 10.1038/s41598-026-51498-8 PMC13333833 42086696

[62] M. Guzman , V. Udoetuk , I. Lee , et al., “Abstract 2369 Structural Variation of PolyUb Cargos Mediates Protein Condensate Potency Abstract 2370 RCSB Protein Data Bank : Updated Resources and New Developments Driving Research and Education in the Biosciences Abstract 2376 Development of a Robust Gel Filtration Protocol That Significantly Minimizes Precipitation During Refolding of Chemically‐,” Journal of Biological Chemistry 302, no. 5 (2026): 112700, 10.1016/j.jbc.2026.112700.

[63] S. P. Jena , P. Nayak , M. A. Adithyan , et al., “Computational Design of Immunogenic Peptide—Ligand Conjugates for Targeted Therapy Against Nipah Virus Infection,” Frontiers in Bioinformatics 6 (2026): 1846380, 10.3389/fbinf.2026.1846380. PMC13454029 42577232

[64] M. Ahamed , M. Km , and S. Ca , “Identification of Potential Anti‐Inflammatory Phytochemicals From Leucas Aspera Using a Computer‐ Aided Drug Design Approach,” International Journal of Medical and Pharmaceutical Sciences 2, no. 8 (2026): 534–541.

[65] J. Uddin , S. Hussain , E. Wahid , S. Tulin , M. Siad , and K. Jannatul , “In Silico DFT Study, Molecular Docking Interactions, Molecular Dynamics and Toxicological Assessment of Selected Organochlorine‐ Based Pesticides,” In Silico Pharmacology 14 (2026): 201. 10.1007/s40203-026-00712-6 PMC13427688 42542631

